# Pregnane X receptor protects against age-related bone loss in males via PI3K/Akt-mediated inhibition of apoptosis

**DOI:** 10.1038/s41420-025-02797-y

**Published:** 2025-11-07

**Authors:** Shangzhi Li, Yu Xu, Wenpeng Xu, Dingxin Zhang, Xiangyu Lin, Peijie Hu, Haipeng Si

**Affiliations:** 1 Department of Orthopedics, Qilu Hospital (Qingdao), Cheeloo College of Medicine, Shandong University, Qingdao, China; 2 Key Laboratory of Qingdao in Medicine and Engineering, Department of Orthopedics, Qilu Hospital (Qingdao), Cheeloo College of Medicine, Shandong University, Qingdao, China; 3 Department of Orthopedics, Qilu Hospital, Cheeloo College of Medicine, Shandong University, Jinan, China; 4 University of Health and Rehabilitation Sciences, Qingdao, China; 5 Department of Medical Experimental Center, Qilu Hospital (Qingdao), Cheeloo College of Medicine, Shandong University, Qingdao, China

**Keywords:** Osteoporosis, Apoptosis

## Abstract

The pregnane X receptor (Pxr) regulates metabolism and inflammation, but its roles in bone homeostasis remain elusive. This study demonstrates that Pxr deficiency in bones induces osteoporotic phenotypes, with reduced trabecular bone mass, impaired osteogenesis, increased inflammation, and apoptosis. RNA sequencing reveals downregulation of the PI3K/Akt signaling pathway in Pxr-deficient bones, a key pathway linked to cell survival and differentiation. In vitro, primary bone marrow mesenchymal stem cells (BMSCs) with Pxr deficiency exhibited inhibited antioxidant enzyme activity, elevated intracellular reactive oxygen species level, activated pro-inflammatory cytokines, suppressed PI3K/Akt pathway, enhanced apoptosis, and decreased osteogenic differentiation. Conversely, Pxr overexpression in BMSCs from aged mice restores PI3K/Akt activation, mitigates apoptosis, and rescues osteogenic differentiation, with these multidirectional beneficial effects abrogated by a PI3K/Akt inhibitor. Moreover, both genetical overexpression of Pxr and pharmacological activation of Pxr improve bone quality in aged mice. These findings identify Pxr as a key regulator of bone homeostasis via the PI3K/Akt pathway, suggesting Pxr as a potential treatment target for age-related bone loss.

## Introduction

Skeletal health is maintained by the delicate balance between osteoblast-mediated bone formation and osteoclast-mediated bone resorption. This process is tightly regulated by signaling pathways and transcription factors systemicly [1, 2]. Disruption of osteoblast differentiation or excessive osteoblast apoptosis can lead to reduced trabecular bone mass and osteoporotic phenotypes. These features are hallmarks of age-related osteoporosis [3, 4]. Nuclear receptors (NRs), such as the vitamin D receptor and peroxisome proliferator-activated receptor γ (PPARγ), are established regulators of osteoblast differentiation and bone marrow stromal cell (BMSC) fate decision [5, 6].

The pregnane X receptor (Pxr), encoded by the gene *Nr1i2*, is a member of the NRs superfamily and functions as a ligand-activated transcription factor. Pxr is renowned for its pivotal roles in regulating xenobiotic and endogenous metabolism, as well as inflammatory responses [7]. Pxr was well identified for its role in hepatic detoxification, where it induces cytochrome P450 enzymes [8, 9]. Recently, Pxr has been proved to be implicated in broader physiological processes, including glucose and lipid homeostasis, bile acid metabolism, and immune modulation [8–11]. Notably, recent studies reported that Pxr represses osteoblast differentiation through the regulation of Hedgehog (Hh) and nuclear factor kappa B (NF-κB) signaling pathways [12, 13]. This finding suggests a potential role for Pxr in bone homeostasis. Despite its well-characterized roles in metabolic organs (e.g., liver and intestine) [14–16], the molecular mechanisms through which Pxr influences osteogenesis, inflammation, and oxidative stress in BMSCs, the progenitors of osteoblasts, remain largely unexplored.

Emerging evidence links Pxr to the regulation of oxidative stress and apoptosis via conserved signaling axes. For instance, Pxr activation protects against cisplatin-induced acute kidney injury by mitigating oxidative stress and apoptosis. This protective effect is mediated through the phosphatidylinositol 3-kinase-protein kinase B (PI3K-Akt) pathway, which acts as a key regulator of cell survival and differentiation [17, 18]. In macrophages, Pxr promotes M2 polarization and reduces inflammatory responses through its interaction with signal transducer and activator of transcription 6 (STAT6) [19]. This observation highlights Pxr’s anti-inflammatory potential. In the context of bone biology, excessive reactive oxygen species (ROS) and pro-inflammatory cytokines (e.g., tumor necrosis factor α (TNF-α), interleukin 1β (IL-1β)) disrupt BMSC function and induce BMSC apoptosis [20, 21]. These effects collectively contribute to bone loss. Given that Pxr modulates the activity of antioxidant enzymes (e.g., superoxide dismutase (SOD), glutathione peroxidase (GSH-PX)) and regulates inflammatory pathways in other tissues [22, 23], it is plausible that Pxr acts as a critical node for balancing osteogenic differentiation, inflammation, and oxidative stress in BMSCs. A critical knowledge gap exists regarding whether and how Pxr regulates osteogenesis and bone homeostasis, especially in aged bones. Although previous studies have demonstrated that Pxr deficiency impairs osteoblast differentiation in vitro [24], the downstream signaling cascades that link Pxr to cell survival and stress responses in bone remain undefined. Moreover, while Pxr agonists have been demonstrated to ameliorate metabolic and inflammatory disorders [25–27], their effects on bone microstructure and age-related osteoporosis remain elusive.

This study aims to investigate the role of Pxr in maintaining bone homeostasis. We posit that Pxr preserves osteogenic function of BMSCs by enhancing their antioxidant capacity, suppressing the expression of pro-inflammatory cytokines, and activating pro-survival signaling pathways. To address this, we utilized adeno-associated virus (AAV) delivering short hairpin RNAs (shRNAs) to generate mice with *Nr1i2* knockdown locally in bones. RNA sequencing was performed to identify Pxr-regulated pathways. Primary BMSCs with *Nr1i2* knockdown were used in vitro to dissect the mechanisms underlying Pxr-mediated osteogenic differentiation and responses to oxidative stress. Additionally, we evaluated the therapeutic potential of pregnenolone 16α-carbonitrile (PCN), a Pxr agonist, as well as the Pxr local overexpression in bones, in improving bone quality in aged mice. By integrating in vivo, in vitro, and translational approaches, this study indicates Pxr as a novel regulator of bone homeostasis. Pxr is found to exert its roles through the PI3K-Akt axis, which coordinates osteogenesis, inflammation, and oxidative stress. Our findings provide new insights into the pathogenesis of age-related bone loss and validate Pxr as a promising therapeutic target for the treatment of age-related osteoporosis.

## Results

### Pxr deficiency induces bone loss in mice

To explore the possible roles of Pxr in bone homeostasis, we first examined Pxr protein expression levels in old (22-month-old) and young (4-month-old) mice. As expected, the old mice exhibited a severe osteoporotic phenotype when compared to young mice. Specifically, femora from old mice showed significantly decreased trabecular bone mineral density (Tb.BMD), trabecular bone volume fraction (Tb.BV/TV), trabecular number (Tb.N) and trabecular thickness (Tb.Th), cortical bone thickness (Ct.Th) alongside an increasement in trabecular separation (Tb.Sp) in femora, as compared to that in young ones (Fig. S1A, B). Notably, Pxr protein expression in the femora of old mice was significantly lower than that in young mice (Fig. S1C, D). Concurrently, the expression of osteogenesis-related protein osterix (Sp7), alkaline phosphatase (Alp), and osteocalcin (Ocn) was found to be significantly downregulated in aged mice (Fig. S1C, D).

**Fig. 1:**
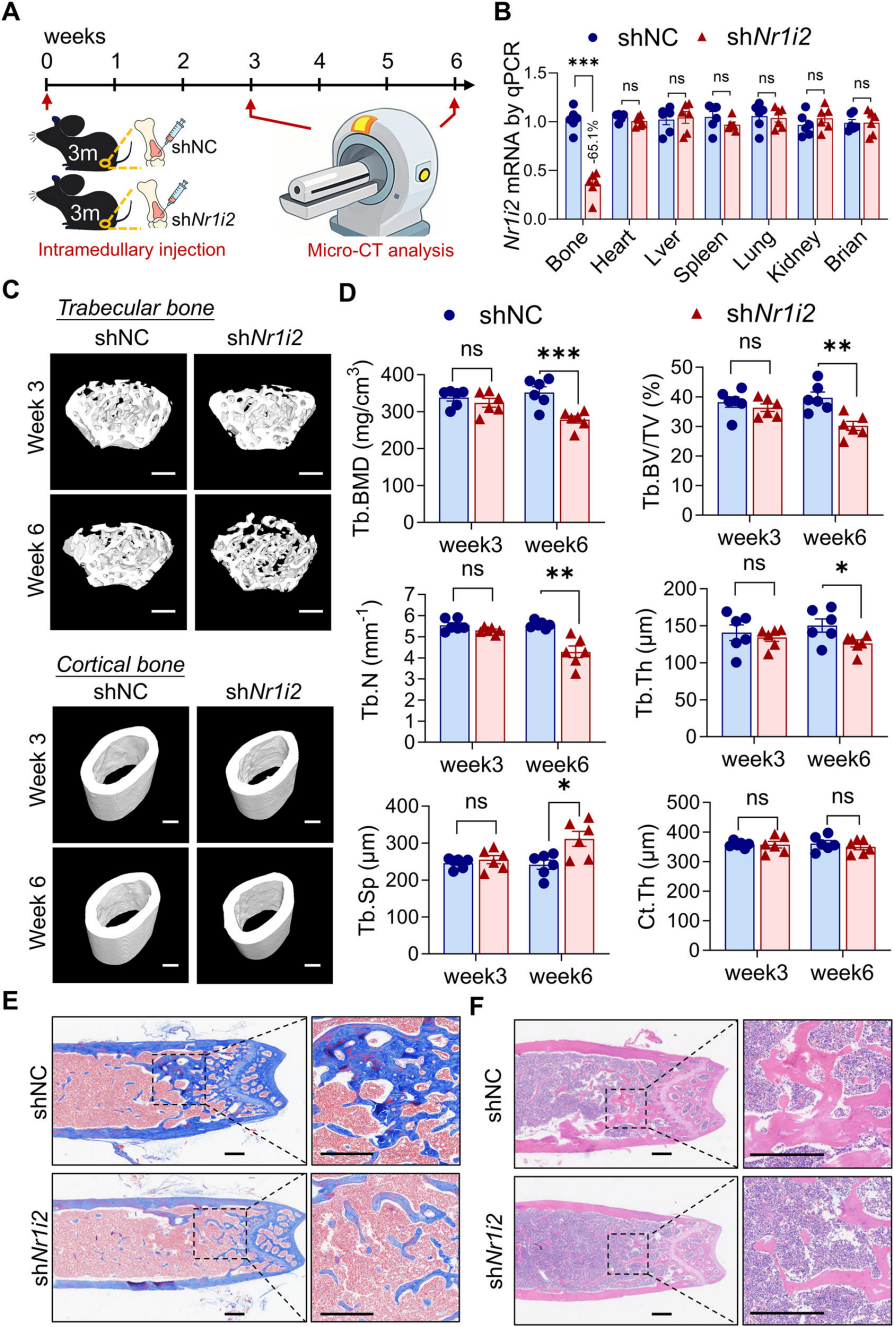
Pxr insufficiency in bones impaired bone quality in mice. **A** Schematic diagram of AAV intramedullary injection and bone quality assessment experimental design. **B** qPCR for *Nr1i2* mRNA expression in different tissues at week 6 post AAV intramedullary injection. **C**, **D** Micro-CT analysis of Tb.BMD, Tb.BV/TV, Tb.N, Tb.Th, Tb.Sp, and Ct.Th in distal end and mid-shaft of femora at week 3 and 6 post AAV shNC and sh*Nr1i2* intramedullary injection, respectively. Histological analysis of femora by masson (**E**) and H&E (**F**) staining at week 6 post AAV intramedullary injection. Data were means ± s.e.m. n = 6 mice in each group. ns *p* > 0.05, *p < 0.05, **p < 0.01, ***p < 0.001 by t-test. Scale bars: 500 μm (**C**, **E**, **F**).

To further investigate whether Pxr contributes to bone homeostasis, we performed intramedullary injections of adeno-associated virus (AAV) into the femora of 3-month-old mice. The AAV constructs either contained short hairpin RNA (shRNA) targeting *Nr1i2* (sh*Nr1i2*), the gene encoding Pxr, or a non-targeting sequence as a control (shNC). Micro-CT analysis was conducted 3 and 6 weeks after AAV injection, respectively (Fig. 1A). Over the 6-week experimental period, there was no significant difference in body weight between the two groups (Fig. S2A), indicating that intramedullary administration of AAVs did not affect the overall health of the mice. We also confirmed exclusive knockdown of *Nr1i2* in bone tissues (Fig. S2B and Fig. 1B). At 6 weeks post-injection, femora from mice treated with AAV-sh*Nr1i2* showed significantly decreased Tb.BMD, Tb.BV/TV, Tb.N, and Tb.Th, along with a significant increase in Tb.Sp, when compared to the control group treated with AAV-shNC. However, Ct.Th remained unaffected by *Nr1i2* knockdown (Fig. 1C, D). Consistent with these micro-CT findings, histological analysis revealed that *Nr1i2* knockdown in the femora also led to reduced collagen deposition and a lower number of trabeculae (Fig. 1E, F). Collectively, these results suggest that Pxr is involved in the pathogenesis of osteoporosis.

### Pxr knockdown alters the transcriptome profile n murine bones

**Fig. 2:**
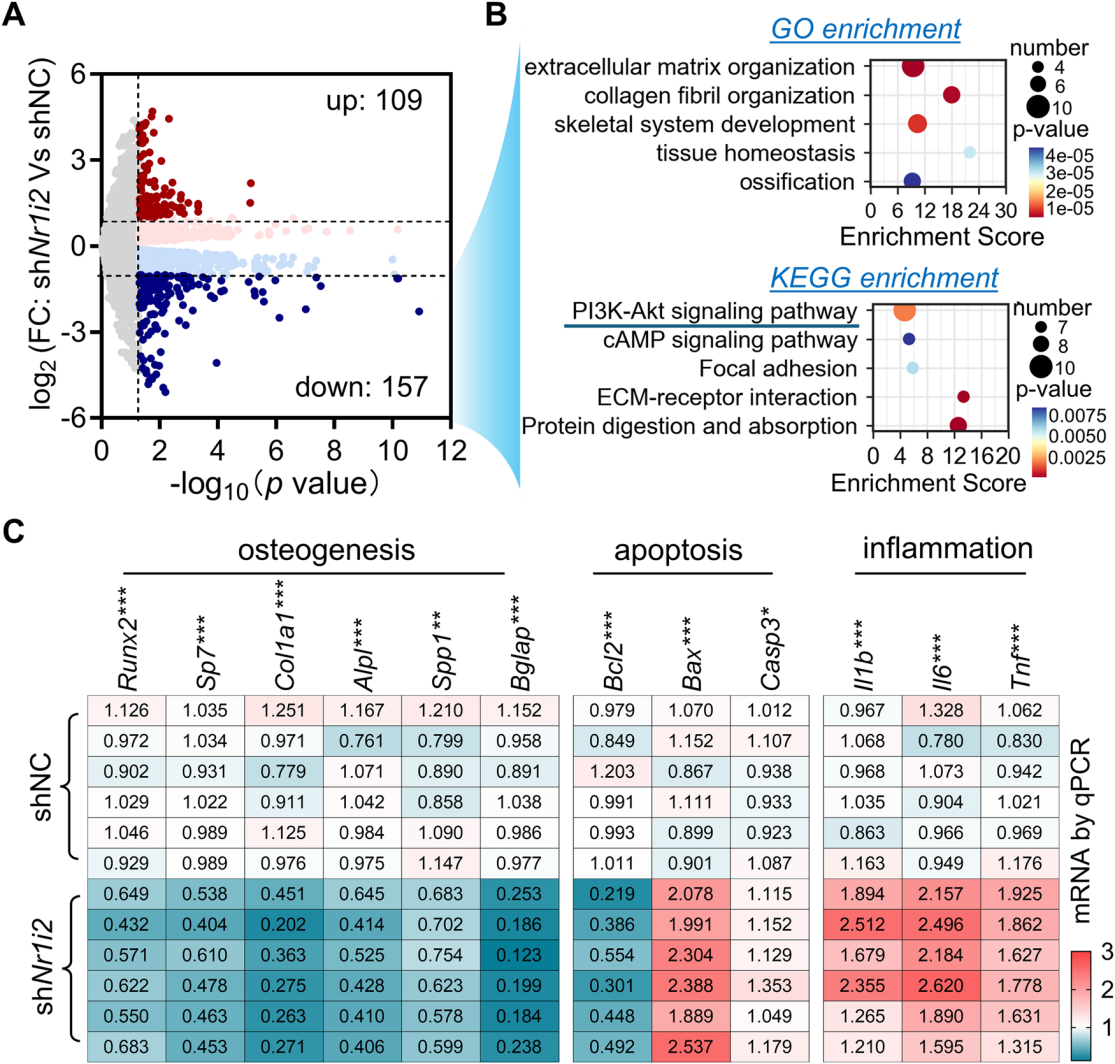
The effect of Pxr deficiency on the transcriptome in bones in mice. **A** The volcano plot showed the DEGs by RNA-Seq in the femora with *Nr1i2* knockdown as compared to that of shNC as control at week 6 post AAV intramedullary injection. **B** GO and KEGG enrichment analysis of the 157 downregulated DEGs as shown in (**A**). **C** qPCR for osteogenesis-, apoptosis-, and inflammation-related genes expression in the femora with *Nr1i2* knockdown and shNC as control at week 6 post AAV intramedullary injection. The numbers in each cell indicated the mRNA expression level. Pseudo color from blue to red indicates the mRNA expression level from low to high. n = 3 **A**, **B**, or 6 (**C**) mice in each group. *p < 0.05, **p < 0.01, ***p < 0.001 by t-test.

To further elucidate the mechanisms by which Pxr deficiency disrupts bone homeostasis, we performed RNA sequencing (RNA-Seq) on femur tissues isolated from experimental mice. Compared to the control group (treated with AAV-shNC), *Nr1i2* knockdown (via AAV-shNr1i2) resulted in significant changes in gene expression: 157 genes were significantly downregulated, while 109 genes were significantly upregulated (Fig. 2A). Gene Ontology (GO) enrichment analysis was conducted to characterize the biological processes affected by *Nr1i2* knockdown. Relative to the shNC control group, femora with *Nr1i2* knockdown showed significant downregulation of biological processes closely associated with osteogenesis, including *skeletal system development* and *ossification* (Fig. 2B). Additionally, Kyoto Encyclopedia of Genes and Genomes (KEGG) enrichment analysis revealed that multiple signaling pathways were significantly downregulated in *Nr1i2*-knockdown femora, with the phosphatidylinositol 3-kinase (PI3K)-protein kinase B (Akt) signaling pathway being one of the key affected pathways (Fig. 2B). To validate the RNA-Seq findings, we measured the mRNA expression of selected genes in all femur samples using quantitative polymerase chain reaction (qPCR). Consistent with the transcriptomic data, *Nr1i2* knockdown in femora was associated with a significant decrease in the mRNA levels of osteogenesis-related genes, and a significant increase in the mRNA levels of inflammation-related genes, when compared to the shNC control group (Fig. 2C). Furthermore, *Nr1i2* knockdown led to reduced mRNA expression of *Bcl2* (encoding the protein B-cell lymphoma 2, Bcl-2) and increased mRNA expression of *Bax* (encoding the Bcl2-associated X protein, Bax) and *Casp3* (encoding the protein caspase 3) in femora. These changes in *Bcl2*, *Bax*, and *Casp3* expression are indicative of activated cellular apoptosis. Collectively, these results demonstrate that Pxr deficiency in young mice not only impairs osteogenic activity in the femur but also exacerbates local inflammation and cellular apoptosis. These effects may be mediated by the inhibition of the PI3K-Akt signaling pathway.

### Pxr deficiency inhibits osteogenesis differentiation of BMSCs in vitro

**Fig. 3:**
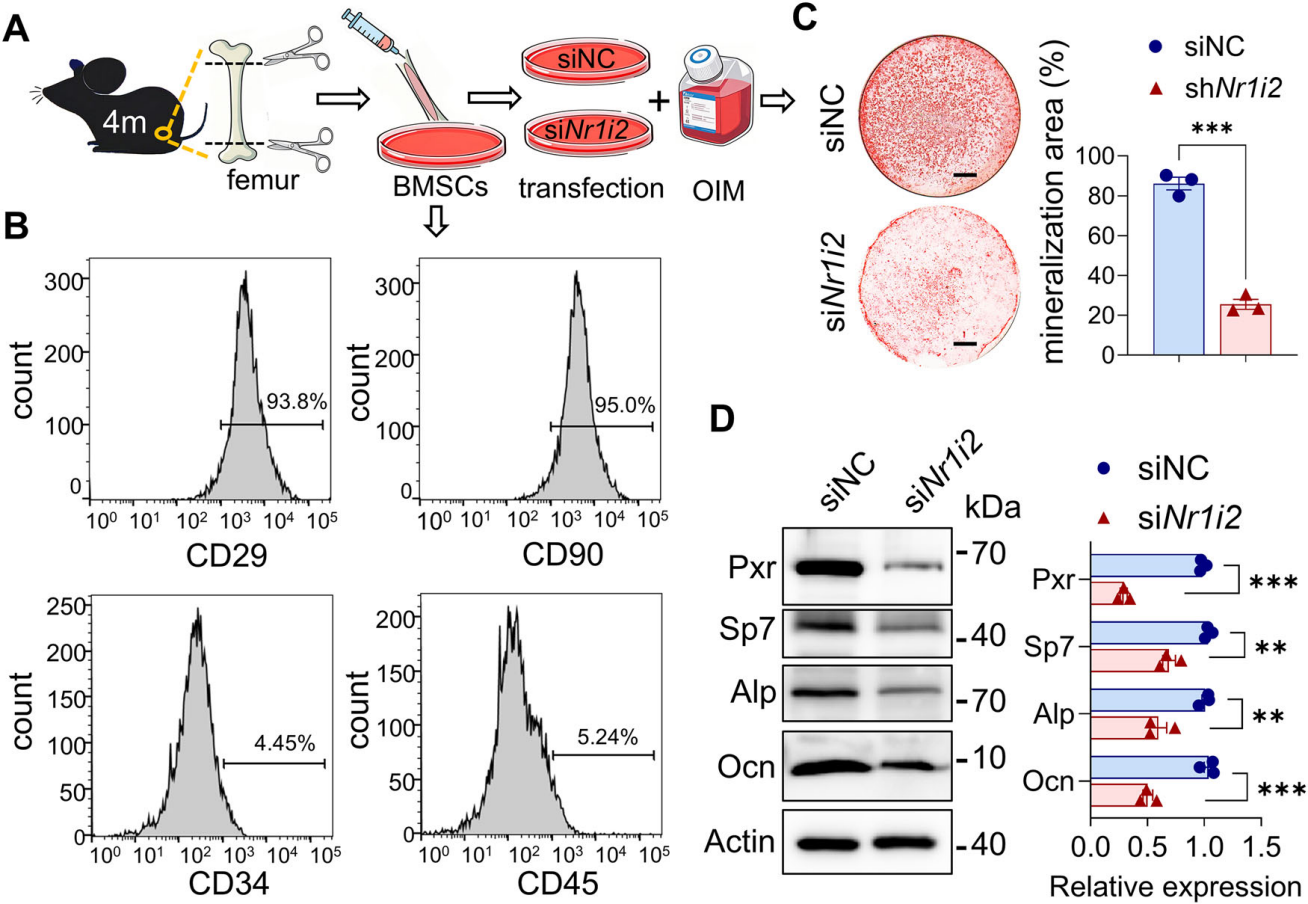
The effect of Pxr insufficiency on osteogenic differentiation in primary BMSCs. **A** Schematic diagram of experimental design showing primary BMSCs extraction and *Nr1i2* knockdown. **B** Flow cytometry analysis of the cell surface immunomarker CD29, CD34, CD45, and CD90 in primary BMSCs. **C** Alizarin Red S staining for mineralization deposits in primary BMSCs with si*Nr1i2* or siNC as control after osteogenic induction. **D** Western blot for Pxr and osteogenic-related proteins in primary BMSCs of different groups after osteogenic induction. Data were means ± s.e.m. n = 3 independent repeats for in vitro experiments. *p < 0.05, **p < 0.01, ***p < 0.001 by t-test. Scale bars: 2.5 mm (**C**).

BMSCs are multipotent stem cells with the capacity for self-renewal and differentiation into osteoblasts. They also play critical roles in bone repair and structural maintenance. To investigate the mechanisms through which Pxr deficiency suppresses osteogenesis, we isolated primary BMSCs from the femora of mice (Fig. 3A). The purity of these primary BMSCs was validated using flow cytometry. Flow cytometric analysis showed negative expression of the hematopoietic lineage cell surface antigens CD34 and CD45, and positive expression of the mesenchymal stem cell markers CD29 and CD90 (Fig. 3B). This marker profile confirmed the identity and purity of the isolated BMSCs. Notably, after successful osteogenic induction of BMSCs (Fig. S3A), the mRNA expression level of *Nr1i2* was significantly higher than that in BMSCs without osteogenic induction (Fig. S3B). This observation suggests that Pxr plays an important role in the osteogenic differentiation of BMSCs. To further assess the function of Pxr in BMSCs, we performed *Nr1i2* knockdown in primary BMSCs in vitro by using small interfering RNAs (siRNAs) targeting the protein-coding sequence (CDS) region of the *Nr1i2* gene (si*Nr1i2*). A non-targeting siRNA (siNC) was used as a control. The efficiency of *Nr1i2* knockdown in primary BMSCs was verified by qPCR (Fig. S3C). Compared to the siNC control group, *Nr1i2* knockdown in primary BMSCs resulted in significant reductions in both cell proliferation and osteogenic differentiation (Fig. S3D and Fig. 3C). Consistent with these phenotypic changes, primary BMSCs with *Nr1i2* knockdown showed significantly downregulated expression of osteogenesis-related protein Sp7, Alp, and Ocn, alongside a decreased Pxr protein level (Fig. 3D). These findings collectively indicate that Pxr deficiency inhibits the osteogenic differentiation potential of BMSCs.

### Pxr insufficiency increased intracellular ROS, inhibited PI3K/Akt pathway and induced apoptosis in primary BMSCs

**Fig. 4:**
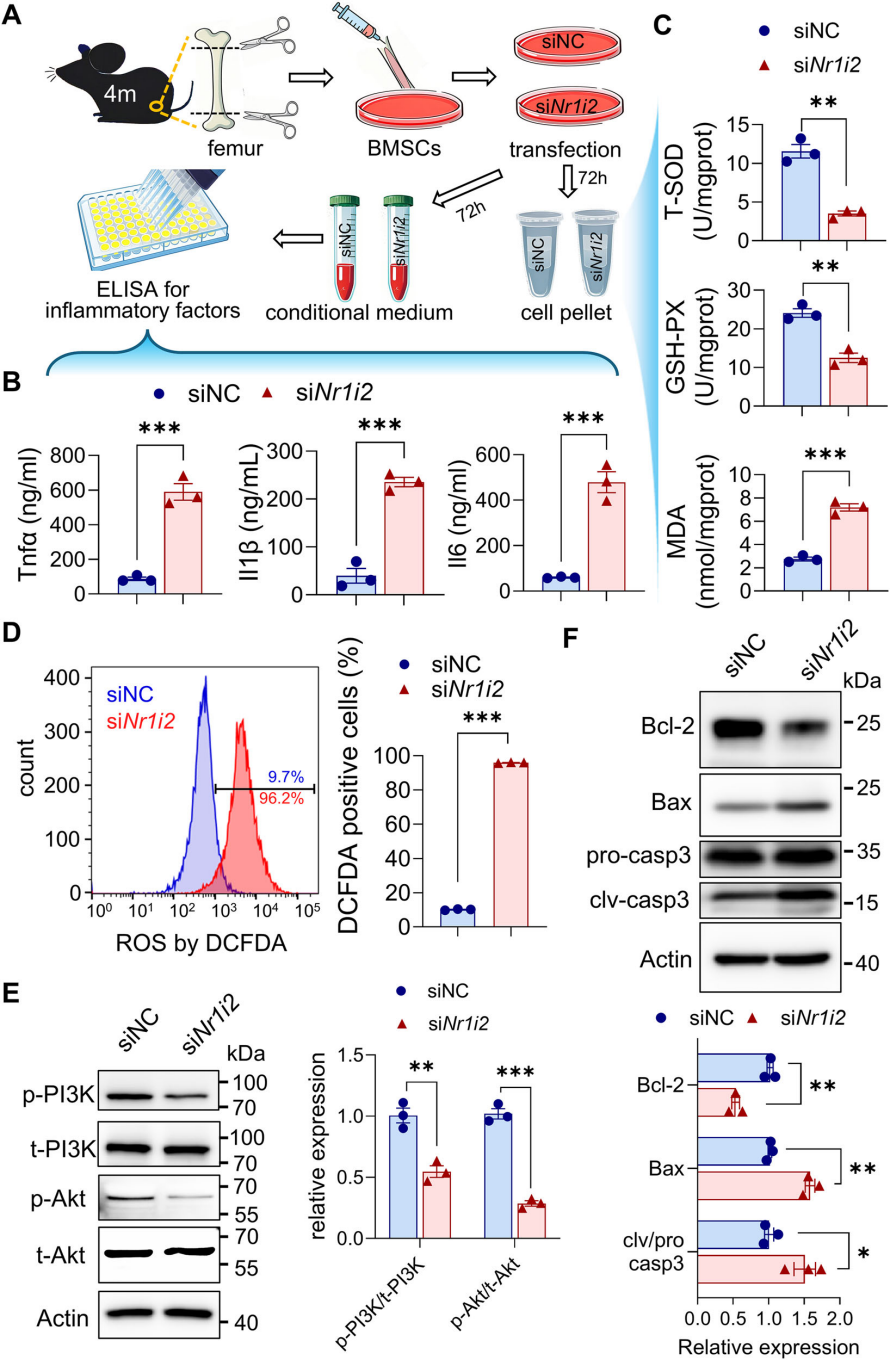
The effect of Pxr deficiency on intracellular ROS, inflammation factors, PI3K/Akt pathway and apoptosis in primary BMSCs. **A** Schematic diagram of experimental design. **B** ELISA for inflammation factors Tnfα, Il1β, and Il6 in the conditional medium collected from *Nr1i2* knockdown primary BMSCs or siNC as control. **C** ELISA for ROS clearance-related enzyme T-SOD, GSH-PX and ROS damage biomarker MDA in *Nr1i2* knockdown primary BMSCs or siNC as control. **D** Flow cytometry analysis of intracellular ROS level by using the fluorescent dye DCFDA. Western blot for PI3K/Akt pathway- (**E**) and apoptosis- (**F**) related proteins in primary BMSCs of different groups. Data were means ± s.e.m. n = 3 independent repeats for in vitro experiments. **p < 0.01, ***p < 0.001 by t-test.

We next asked how Pxr deficiency in primary BMSCs leads to decreased cellular proliferation and osteogenesis differentiation. We found that the inflammation factors, Tnfα, Il1β, and Il6, were significantly upregulated in the conditional medium collected from primary BMSCs with *Nr1i2* knockdown as compared to that of siNC as control (Fig. 4A, B). This result indicates that Pxr deficiency activates inflammatory signaling pathways in primary BMSCs. Next, we assessed oxidative stress status in these cells by measuring the activity of ROS-scavenging enzymes and the level of oxidative damage markers. Relative to the siNC control group, primary BMSCs with *Nr1i2* knockdown exhibited significantly reduced intracellular level total superoxide dismutase (T-SOD) and glutathione peroxidase (GSH-PX), two enzymes capable of eliminating reactive oxygen species (ROS) [28]. In contrast, the level of malondialdehyde (MDA), a well-recognized biomarker of cellular oxidative damage [28], was significantly increased in *Nr1i2*-knockdown BMSCs (Fig. 4C). To directly visualize intracellular ROS accumulation, we used 2’,7’-dichlorofluorescin diacetate (DCFDA), a permeable fluorescent dye that specifically detects intracellular ROS. Flow cytometric analysis revealed a significantly higher percentage of DCFDA-positive (DCFDA⁺) cells in the Nr1i2-knockdown group compared to the siNC control group (Fig. 4D). Collectively, these data demonstrate that Pxr deficiency leads to abnormal accumulation of intracellular ROS in primary BMSCs.

To confirm that oxidative stress is a critical contributor to Pxr’s function in maintaining bone homeostasis, we performed a rescue experiment using the antioxidant γ-L-glutamyl-L-cysteinyl-glycine (GSH). Mice with AAV-mediated Pxr knockdown (AAV-sh*Nr1i2*) were subjected to two treatment regimens: one group received GSH via tail vein injection at a dose of 10 mg/kg, while the other group received an equal volume of normal saline (serving as the vehicle control). Injections were administered twice weekly for a total duration of 6 weeks. Micro-CT analysis was conducted to assess changes in femoral bone structure (Fig. S4A). Compared to the vehicle control group, GSH treatment significantly prevented bone loss in the femora induced by Pxr-knockdown (Fig. S4B, C). Collectively, these data indicate that oxidative stress acts as a key mediator of Pxr’s role in preserving bone homeostasis.

We further investigated whether Pxr deficiency affects the PI3K-Akt pathway and cellular apoptosis. We found that *Nr1i2* knockdown reduced the phosphorylation levels of PI3K and Akt in primary BMSCs, indicating that *Nr1i2* knockdown impairs the activation of the PI3K/Akt signaling pathway (Fig. 4E). In addition, compared to the siNC control group, *Nr1i2* knockdown in BMSCs led to a significant decrease in the expression of the anti-apoptotic Bcl-2. Conversely, the expression of the pro-apoptotic protein Bax and the cleaved (active) form of the apoptosis executor caspase 3 (cleaved caspase 3) was significantly increased (Fig. 4F). These findings are in line with our RNA-Seq results as described above and confirm that Pxr insufficiency leads to inhibition of PI3K/Akt signaling pathway and activation of apoptotic pathway in primary BMSCs.

### Pxr-mediated anti-apoptotic and pro-osteogenic effects in BMSCs depend on the PI3K/Akt pathway

**Fig. 5:**
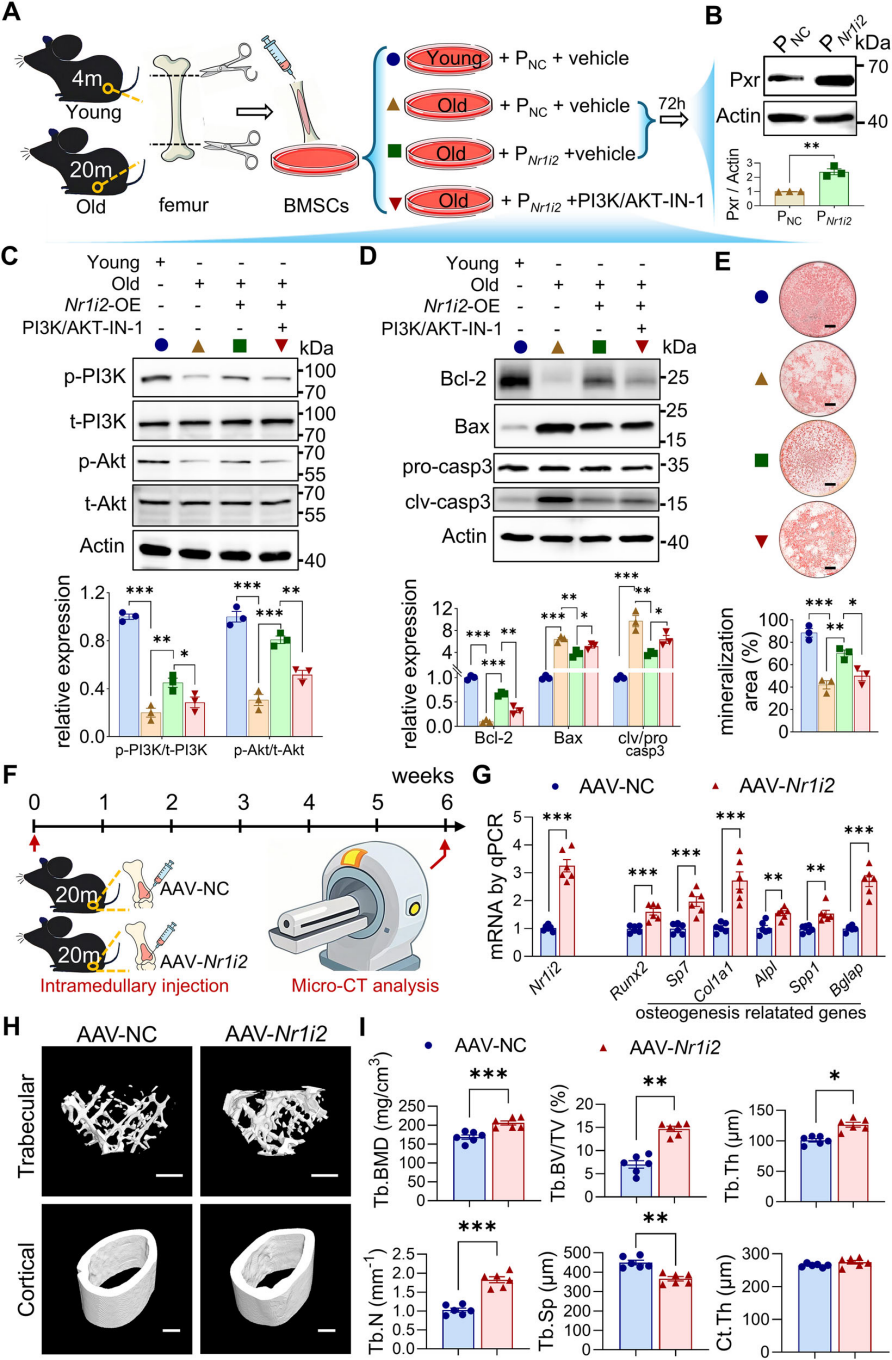
The effect of Pxr overexpression on primary BMSCs and bones from aged mice. **A** Schematic diagram of experimental design for BMSC in vitro experiment. Western blot for Pxr (**B**), PI3K/Akt pathway- (**C**) and apoptosis- (**D**) related proteins in primary BMSCs of different groups. **E** Alizarin Red S staining for mineralization deposits in primary BMSCs of different groups. **F** Schematic diagram of experimental design for AAV-mediated Pxr overexpression in bone in aged (20-month-old) male mice in vivo. **G** qPCR for the mRNA expression level of *Nr1i2* and genes related to osteogenesis in femora of different groups. **H**, **I** Micro-CT analysis of Tb.BMD, Tb.BV/TV, Tb.N, Tb.Th, Tb.Sp, and Ct.Th in distal end and mid-shaft of femora in mice of different groups at week 6 post AAV intramedullary administration. Data were means ± s.e.m. n = 3 independent repeats for in vitro experiments, or 6 mice in each group. *p < 0.01, **p < 0.01, ***p < 0.001 by t-test or ANOVA. Scale bars: 2.5 mm (E), and 500 μm (H).

To verify whether the function of Pxr in BMSCs depends on the PI3K/Akt pathway, we isolated primary BMSCs from young and aged mice. We performed Pxr overexpression in these cells by using the plasmid containing the full-length coding sequence of Mus musculus *Nr1i2* (P_*Nr1i2*_) or empty vector as negative control (P_NC_), and used PI3K/AKT-IN-1, a specific inhibitor of the PI3K/Akt pathway [29], to assess the pathway’s role in mediating Pxr function in BMSCs (Fig. 5A). First, we confirmed successful overexpression of Pxr in primary BMSCs isolated from aged mice (Fig. 5B). Next, we examined the activity of the PI3K/Akt pathway. Compared with BMSCs from young mice, the PI3K/Akt pathway was significantly downregulated in primary BMSCs from aged mice. Pxr overexpression significantly enhanced PI3K/Akt pathway activity in aged BMSCs, while treatment with the inhibitor PI3K/AKT-IN-1 (5 μM) suppressed this Pxr overexpression-induced activation of the PI3K/Akt pathway (Fig. 5C). We further analyzed the apoptotic pathway. Compared with primary BMSCs from young mice, the apoptotic pathway was significantly activated in BMSCs from aged mice. Pxr overexpression inhibited this apoptotic pathway; interestingly, however, the inhibitory effect of Pxr overexpression on the apoptotic pathway was significantly attenuated following treatment with PI3K/AKT-IN-1 (Fig. 5D). Additionally, we evaluated osteogenic differentiation capacity under these conditions. Osteogenic potential was significantly reduced in BMSCs from aged mice compared with those from young mice, and Pxr overexpression restored this osteogenic capacity. Notably, the promotional effect of Pxr overexpression on osteogenesis in aged BMSCs was significantly weakened by the addition of PI3K/AKT-IN-1 (Fig. 5E). Collectively, these results demonstrate that the anti-apoptotic and pro-osteogenic effects of Pxr in BMSCs are dependent on the PI3K/Akt pathway.

### AAV-medicated Pxr overexpression in bones improved bone mass in aged mice

To further validate the role of pregnane X receptor (Pxr) in mitigating age-related bone loss, we investigated the effects of direct Pxr overexpression in the bones of aged mice on bone mass. Two AAV constructs were used: one containing the full-length coding sequence of Mus musculus *Nr1i2* (AAV-*Nr1i2*) to overexpress Pxr, and a negative control virus (AAV-NC). These AAVs were locally injected into the femoral medullary cavity of 20-month-old male mice (Fig. 5F). qPCR confirmed efficient overexpression of *Nr1i2* in bone tissues (Fig. 5G). Consistent with our hypothesis, AAV-mediated *Nr1i2* overexpression in bones significantly upregulated the mRNA levels of key osteogenesis-related genes (Fig. 5G). At 6 weeks post-injection, micro-CT analysis revealed significant improvements in femoral bone mass and structural parameters in the AAV-*Nr1i2* group compared to the AAV-NC control group (Fig. 5H, I). Collectively, these results confirm that Pxr was a potential therapeutic target for the treatment of age-induced bone loss.

### Pxr agonist pregnenolone-16α-carbonitrile (PCN) improved bone mass in aged mice

**Fig. 6:**
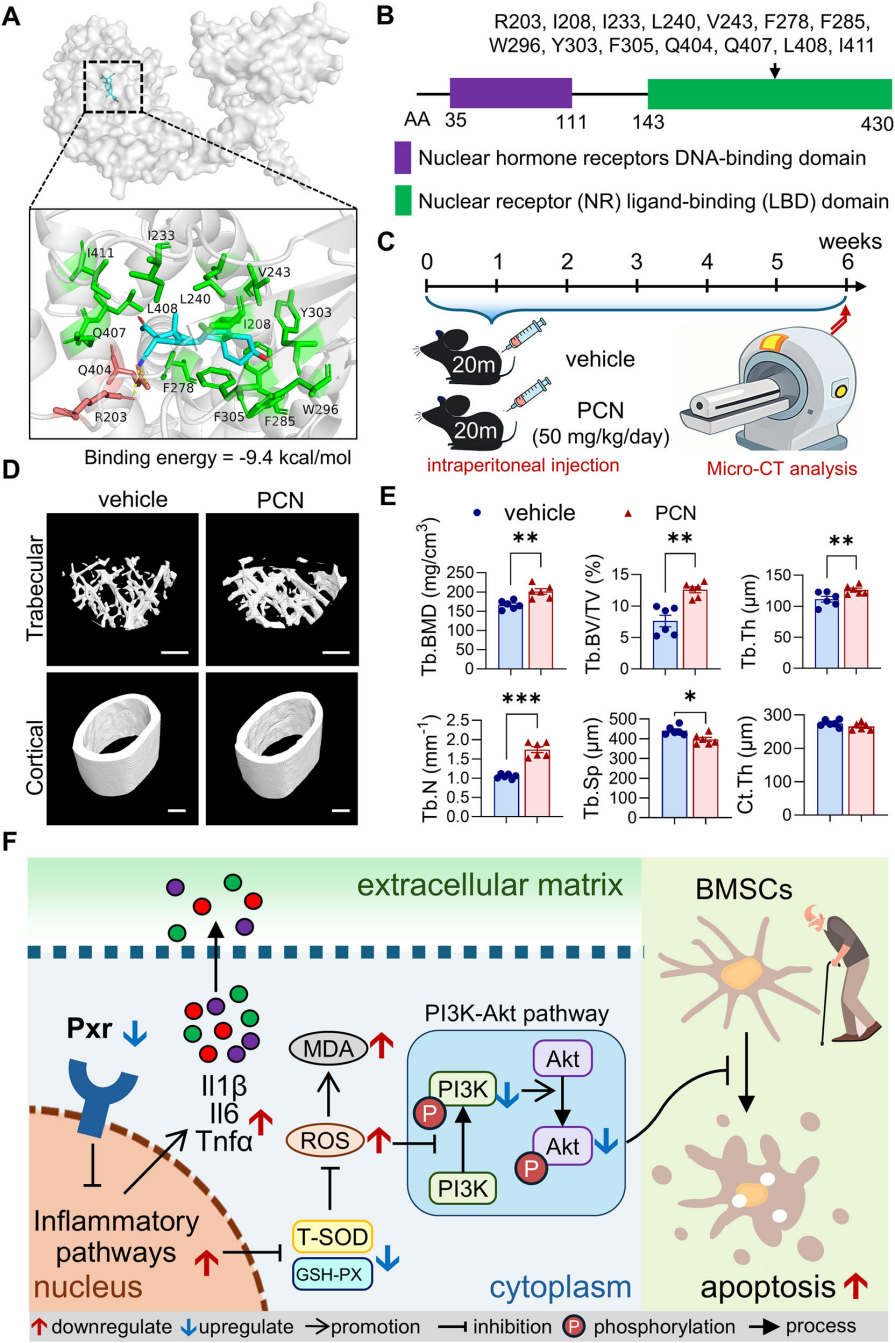
The Pxr agonist PCN rescues age-related osteoporosis in aged male mice. **A** Molecular docking of mus musculus Pxr protein and PCN. The backbone of the Pxr is depicted as gray, while PCN is displayed in cyan. The surrounding residues in the binding pocket are shown in green and brown. **B** Schematic diagram showing the domains of mus musculus Pxr protein and PCN binding regions. **C** Schematic diagram of experimental design showing the PCN administration and assessment of bone quality in aged (20-month-old) mice. **D**, **E** Micro-CT analysis of Tb.BMD, Tb.BV/TV, Tb.N, Tb.Th, Tb.Sp, and Ct.Th in distal end and mid-shaft of femora at week 6 post PCN administration (50 mg/kg/day) or equivalent amount of vehicle as control. **F** Schematic diagram showing the summary of this study. Data were means ± s.e.m. n = 6 mice in each group. *p < 0.05, **p < 0.01, ***p < 0.001 by t-test. Scale bars: 500 μm (**D**).

Although Pxr overexpression exerted highly effective protective effects on old BMSCs and aged bone tissues, this transgenic approach has limitations for practical applications. To enhance the clinical translational potential of our research, we used a pharmacological strategy by investigating the effects of PCN, a well-characterized agonist of murine Pxr [16, 30], on aged mouse bones. Molecular docking analysis between PCN and the Pxr protein revealed that PCN is a high-affinity ligand for murine Pxr, with a binding energy of −9.4 kcal/mol (Fig. 6A). All binding regions of PCN with the Pxr protein were identified to be localized within the nuclear receptor (NR) ligand-binding domain (LBD) of the Pxr protein (Fig. 6B). To evaluate whether PCN could improve bone quality in aged mice, 20-month-old mice were administered either PCN (50 mg/kg) or an equivalent volume of vehicle (as a control) via daily intraperitoneal injections for 6 weeks (Fig. 6C). Notably, PCN treatment significantly improved bone quality in aged mice compared to the vehicle control group. Specifically, PCN administration increased Tb.BMD, Tb.BV/TV, Tb.N, and Tb.Th, while reducing Tb.Sp (Fig. 6D, E). These findings suggest that enhancing Pxr function in aged mice via pharmacological activation represents a novel therapeutic strategy for the treatment of age-related osteoporosis.

## Discussion

The present study identifies a critical role for Pxr in maintaining bone homeostasis. Specifically, we demonstrate that Pxr deficiency in mice promotes inflammation and oxidative stress, inhibits the PI3K/Akt signaling pathway, induces cellular apoptosis, and impairs osteogenic differentiation. Collectively, these effects disrupt bone metabolic balance and ultimately lead to the development of osteoporotic phenotypes (Fig. 6D). These findings establish Pxr as a novel regulator of bone health.

### Pxr as a key mediator of osteogenesis

Previous studies have primarily focused on Pxr’s roles in metabolic and inflammatory regulation, but its function in bone biology has remained elusive. Our results show that Pxr expression declines with aging in mice, paralleling the reduction in osteogenic markers (Sp7, Alp, Ocn) and the emergence of osteoporotic traits, such as decreased trabecular bone mass and microstructural deterioration. Genetic knockdown of *Nr1i2* in young mice recapitulated these age-related bone changes, while direct *Nr1i2* overexpression in bones rescued aging-induced bone loss. These findings confirm a causal relationship between Pxr and bone hemostasis. Notably, while *Nr1i2* knockdown was confirmed as early as 3 weeks post-AAV intramedullary injection, significant trabecular bone loss was only observed at 6 weeks. This temporal lag likely reflects the slow kinetics of bone remodeling: osteogenic differentiation and matrix mineralization are protracted processes that require several weeks to translate into measurable changes in bone mass [31, 32]. At 3 weeks, Pxr deficiency may have induced subtle impairments in BMSC’s function. However, these early defects were insufficient to exceed the threshold for homeostatic compensation in the bone microenvironment. In contrast, prolonged Pxr depletion over 6 weeks progressively compromised osteoblast formation and function, leading to cumulative deficits in trabecular bone mass. Besides, Ct.Th remained unaffected by *Nr1i2* knockdown, overexpression or administration of Pxr agonist PCN over 6 weeks. This differential response between trabecular and cortical bone may be attributed to inherent differences in their turnover dynamics [33]. Trabecular bone, characterized by a high surface area-to-volume ratio and rapid remodeling rate, is more sensitive to acute disruptions in osteoblast function, making it vulnerable to early changes in Pxr-dependent osteogenesis. In contrast, cortical bone is stabilized by mechanical loading and exhibits a much slower remodeling rate [33, 34]. As a result, it may require longer-term or more severe Pxr deficiency to manifest detectable structural changes. Additionally, Pxr may primarily regulate osteogenesis in the bone marrow microenvironment. This localization would have a more pronounced impact on trabecular bone. In contrast, cortical bone in the diaphysis is more heavily influenced by distinct mechanical signaling pathways and developmental programs, which may be less dependent on Pxr activity [35, 36].

### Specificity concerns of AAV local administration by intramedullary injection in bones

Intramedullary injection of AAVs is a well-established method for gene knockdown or overexpression in bone marrow cells in rodents according to previous publications [37]. We have evaluated the specificity of local AAV administration using qPCR and found that the mRNA expression level of *Nr1i2* was significantly decreased in bones at both week 3 and week 6, while remaining unchanged in other tissues. We hypothesize several possible reasons for this phenomenon, even though the AAVs were not designed to be bone specific. First, the AAV serotype used in our study is AAV9, which is known to exhibit intrinsic tropism in infecting bone-derived cells [38]. Second, intramedullary injections allow AAVs to concentrate locally in bone marrow and bone tissues, preferentially transducing resident cells in these sites. Third, the limited volume of the AAV solution (10 µl, with 10^10 ^v.g.) used for the femoral intramedullary injection, together with the dense nature of bone tissue may restrict viral diffusion systematically. In sum, the combined effects of the injection method (localized delivery) and the serotype’s tropism would result in extremely low infection rates in non-bone tissues, even if a small amount of virus escapes from bone cavity. Such off-target infections are unlikely to maintain sustained viral activity, further minimizing non-bone gene modulation.

### Pxr regulates the oxidative-inflammatory-apoptotic axis in BMSCs

A notable observation from our study was the exacerbation of inflammatory and apoptotic pathways in *Nr1i2*-knockdown bone tissues. Specifically, Pxr-deficient BMSCs showed increased production of pro-inflammatory cytokines, along with elevated intracellular ROS levels. Concurrently, these cells exhibited reduced activity of key antioxidant enzymes. This dysregulated oxidative-inflammatory microenvironment likely drives cellular apoptosis. Supporting this, Pxr-deficient BMSCs displayed upregulated expression of the pro-apoptotic protein Bax and the cleaved (active) form of caspase 3, alongside downregulated expression of the anti-apoptotic protein Bcl-2. Oxidative stress is a well-documented contributor to age-related bone loss, as excess intracellular ROS impairs stem cell self-renewal and differentiation capacity while promoting osteoblast apoptosis [39–41]. Our rescue experiment further validated the role of ROS in Pxr-mediated bone homeostasis. The treatment with the antioxidant GSH significantly prevented bone loss induced by *Nr1i2* knockdown. This finding confirms that ROS accumulation is a critical downstream mediator of Pxr deficiency in bone. Our study establishes a direct link between Pxr and the oxidative-inflammatory-apoptotic pathological axis in BMSCs. These results suggest that Pxr functions as a redox-inflammatory sensor in BMSCs, where it helps maintain redox balance, suppress excessive inflammation, and thereby safeguard bone cells against oxidative damage and apoptosis.

### The PI3K/Akt pathway is a downstream mediator of Pxr’s protective effects

RNA-Seq identified the PI3K/Akt signaling pathway as a key downstream target of Pxr in bone. Pxr deficiency suppressed the phosphorylation of PI3K and Akt, the activation of which is critical for regulating cell proliferation, survival, and differentiation [42]. Functional validation experiments revealed that Pxr overexpression in BMSCs from aged mice activated the PI3K/Akt pathway, inhibited apoptotic signaling, and promoted osteogenic differentiation. Notably, these beneficial effects of Pxr overexpression were abolished by treatment with a PI3K/Akt inhibitor. This finding provides direct evidence that the PI3K/Akt pathway is a critical mediator of Pxr’s regulatory effects on BMSC survival and osteogenic potential. These results align with previous reports linking PI3K-Akt activation to enhanced osteoblast formation and apoptosis inhibition [18]. It underscores the therapeutic potential of Pxr in boosting this pro-survival pathway to preserve bone mass.

### Therapeutic implications and future directions

The identification of Pxr as a regulator of bone homeostasis via the oxidative stress-inflammation-PI3K/Akt axis introduces a new therapeutic strategy for osteoporosis. Current osteoporosis treatments primarily focus on inhibiting bone resorption (e.g., bisphosphonates) or modestly enhancing bone formation (e.g., parathyroid hormone analogs) [43, 44]. In contrast, Pxr agonists may represent a novel class of agents that simultaneously promote osteogenesis, reduce inflammation, and mitigate oxidative stress, which addresses multiple pathological mechanisms of bone loss. Notably, despite the low Pxr protein levels in the bones of aged mice, treatment with the Pxr agonist PCN still significantly improved bone quality. This finding confirms that Pxr is a potent osteogenic target. The underlying mechanism for this efficacy is supported by molecular docking analyses, which demonstrated strong binding affinity between PCN and murine Pxr. This high-affinity interaction implies that even with low Pxr protein expression, PCN-mediated activation of Pxr is sufficient to trigger downstream signaling pathways involved in bone homeostasis.

In the present study, our investigations into age-related bone loss were restricted to male mice. The pathogenesis of age-related osteoporosis differs significantly between males and females. With aging, women experience two phases of bone loss: an early accelerated phase in the first decade after menopause, driven by rapid estrogen decline that directly enhances bone resorption without sufficient compensatory bone formation; and a late slow phase involving persistent bone loss, linked to estrogen deficiency-induced abnormalities in calcium homeostasis and increased parathyroid hormone secretion, both of which promote bone resorption. In contrast, men lack a postmenopausal-like rapid bone loss phase, exhibiting only a continuous slow phase of bone loss [45, 46]. Though age-related accumulation of senescent/apoptotic cells contributes to age-related osteoporosis in both sexes, age-related osteoporosis in women is more directly influenced by hormonal factors. Given these pathogenic differences, we focused exclusively on male osteoporosis as our research subject to ensure the rigor and accuracy of our conclusions.

Several questions remain to be addressed in future study. First, as described above, while the study focused on age-related osteoporosis in males, the roles of Pxr in other types of osteoporosis (e.g., postmenopausal osteoporosis) awaits to be explored. Second, the study has demonstrated the regulatory roles of Pxr on oxidative stress and apoptosis in BMSCs, the role of Pxr in bone fracture healing awaits to be investigated, as BMSC is crucial for the fracture healing process [47]. Finally, since the BMSCs also possess the capacity for chondrogenic differentiation, the role of Pxr in regulating BMSC chondrogenic differentiation, and its potential therapeutic value in alleviating osteoarthritis is worthy to be explored.

In summary, this study indicates Pxr as a crucial regulator of bone homeostasis. Pxr integrates inflammatory signaling, oxidative stress responses, apoptotic pathway and osteogenic differentiation processes in BMSCs via PI3K/Akt pathway. Pxr deficiency disrupts this regulatory network, perturbing bone metabolic balance and ultimately leading to bone loss. In contrast, genetical overexpression of Pxr or pharmacological activation of Pxr improves bone quality in aged mice. The findings not only deepen our understanding of the molecular mechanisms underlying osteoporosis but also highlight Pxr as a promising therapeutic target for developing novel treatments for age-related bone diseases.

## Materials and methods

### Animals

Animal experiments were conducted strictly with animal care and experimental protocols under ethics approval (KYDWLL-202427) from the *Animal Use and Care Committee* at Qilu Hospital (Qingdao) of Shandong University. C57BL/6 J male mice were purchased from the Beijing Vital River Laboratory Animal Technology Co., Ltd. All mice were housed under specific-pathogen-free (SPF) conditions, with a 12-h light/dark cycle and a constant temperature of 25 °C. The age-matched male mice were blinded randomly assigned into different groups.

### Adeno-associated virus (AAV)-based *Nr1i2* knockdown or overexpression in vivo

AAV-mediated *Nr1i2* gene knockdown or overexpression in femora was performed according to well-established protocols [48]. Briefly, AAV-packaged shRNA (5′-CCA CTC ATG CAA GAG TTA TTT-3′) targeting mus musculus *Nr1i2* gene (pAAV-U6-sh*Nr1i2*-CMV-MCS-WPRE, hereafter referred to as sh*Nr1i2*) or non-targeting sequence (5′-CCT AAG GTT AAG TCG CCC TCG-3′) as knockdown negative control (pAAV-U6-shNC-CMV-MCS-WPRE, shNC), and AAVs containing the full-length coding sequence of mus musculus *Nr1i2* gene (NCBI ID: NM_010936.3, pAAV-CMV-*Nr1i2*-WPRE, AAV-*Nr1i2*) or a vector containing a null sequence (pAAV-CMV-MCS-WPRE, AAV-NC) as the overexpression negative control were purchased from QBiO Technology (Shanghai) Co., Ltd. A total amount of 1 × 10^10 ^v.g. AAVs in 10 μL normal saline were locally injected into the bilateral femoral marrow cavity of the femora by using a syringe equipped with 29 Gauge needle in each male C57BL/6 J mouse. The femora were collected at week 3 or 6 after AAV local injection for further analysis. The gene knockdown efficiency was evaluated by qPCR assay.

### Real-time quantitative PCR (qPCR)

Total RNA was extracted from cells or tissues by using FastPure cell/tissue total RNA isolation kit V2 (RC112, Vazyme, Nanjing, Jiangsu, China) according to the manufacturer’s instructions. The concentrations of RNA were determined by using a bio-spectrometer (Eppendorf). After the RNA was reverse-transcribed into cDNA by using High Capacity cDNA Reverse Transcription Kit (4374966, Thermo, Rockford, IL, USA) according to the manufacturer’s instructions, qPCR was done by using TB Green Premix (RR420A, TaKaRa, Kusatsu, Shiga, Japan) and primers shown in Table S1. *Gapdh* was used as a reference gene. The relative fold of gene expression was calculated by the 2^−ΔΔCT^ formula.

### Micro-CT assessment

The femora of mice were collected and fixed with 4% paraformaldehyde (PFA, P1110, Solarbio, Beijing, China) at 4 °C for 48 h and subsequently stored in 70% ethanol at 4 °C until analysis. Micro-CT was done using a Quantum GX microCT scanner (PerkinElmer, Waltham, MA, USA) according to the manufacturer’s pre-setting protocols. The 3D reconstruction images of the distal and mid-shaft femora were obtained. The trabecular bone mineral density (Tb.BMD), trabecular bone volume fraction (Tb.BV/TV), trabecular number (Tb.N), trabecular thickness (Tb.Th), trabecular separation (Tb.Sp), and average cortical thickness (Ct.Th) were quantified by using the accompanied Analyze 12.0 software (PerkinElmer, Waltham, MA, USA).

### Histological staining

The femora of mice were collected and fixed with 4% PFA (P1110, Solarbio, Beijing, China) at 4 °C for 48 h, and subsequently decalcified in 10% ethylenediamine tetraacetic acid (EDTA, pH 7.4, 10009617, Sinopharm, Shanghai, China) for two weeks at 4 °C. Then the femora were embedded in paraffin for sections after being dehydrated by a series of ethanol with concentrations of 70%, 80% 90%, and 100%, and clearing treatment by xylene. Sections with a thickness of 5μm were obtained by using a microtome (HistoCore BIOCUT, Leica, Wetzlar, Germany) and subjected to Hematoxylin and Eosin (H&E) and Masson staining. Images were obtained by using a microscope (Ts2R-FL, Nikon, Shinagawa-ku, Tokyo, Japan).

### Western blot

The total proteins were extracted by using RIPA lysis buffer (89901, Thermo, Rockford, IL, USA) with presence of a cocktail of protease and phosphatase inhibitors (78443, Thermo, Rockford, IL, USA) according to the manufacturer’s instructions. Protein concentrations were determined by using the BCA assay (23225, Thermo, Rockford, IL, USA). A total of 50 μg protein samples were separated by SDS-PAGE and subsequently electrotransferred onto a PVDF membrane (ISEQ00010, Millipore Sigma, MA, USA). After blocking the membranes with 5% non-fat milk (P0216, Beyotime, Shanghai, China), primary antibodies (Table S2) were added, and the membranes were incubated at 4 °C overnight. The next day, secondary antibodies (Table S2) were applied, and the incubation continued at room temperature for 1 h. Protein bands were detected using a Pierce ECL Western Blotting Kit (32209, Thermo, Rockford, IL, USA) with the Amersham Imager 600 System (General Electric Company, Boston, MA, USA). Band densitometry analysis was conducted using ImageJ software (version 1.52a, NIH, Bethesda, MD, USA) to quantify gray values.

### RNA-sequencing (RNA-Seq)

The femora of mice were collected, quick-frozen by liquid nitrogen, and stored at −80 °C before being sent to Shanghai OE Biotech Co., Ltd for transcriptome sequencing analysis. FPKM (fragments per kilobase of transcript sequence per million base pairs sequenced) is utilized for quantifying the gene expression levels of different samples. A threshold of *p-*value < 0.05 and |log_2_(foldchange)| > 1 was set for significantly differential expression genes (DEGs) between groups. Based on hypergeometric distribution, Gene Ontology (GO) and Kyoto Encyclopedia of Genes and Genomes (KEGG) enrichment analysis for DEGs was conducted using the online tool (https://cloud.oebiotech.com/#/bio/tools).

### Primary BMSCs isolation and validation

Primary BMSCs were extracted from the femoral bone marrow of C57BL/6J mice according to the well-established protocols [49]. Femora from mice were harvested, and both epiphyses were excised. Bone marrow contents were then flushed into 10-cm culture dishes using a 25G needle-equipped syringe, with α-MEM medium (12571063, Gibco, Grand Island, NY, USA) supplemented with 10% fetal bovine serum (FBS, F101, Vazyme, Nanjing, Jiangsu, China) and 1% penicillin-streptomycin (PS, 15140122, Gibco, Grand Island, NY, USA). Cultures were maintained in a 37 °C incubator with 5% CO₂. To remove non-adherent cells, half-volume medium replacement was performed daily until cells reached 90% confluency, at which point they were passaged. For phenotypic characterization, primary BMSCs were harvested and incubated with fluorescently conjugated antibodies against CD29, CD34, CD45, and CD90 (Table S2) in the dark at room temperature for 30 min. After washing with phosphate-buffered saline (PBS), cell samples were analyzed by flow cytometry using a FACS Celesta system (BD Biosciences, Franklin Lakes, NJ, USA).

### BMSCs osteogenic induction

Primary BMSCs were cultured in α-MEM (12571063, Gibco, Grand Island, NY, USA) supplemented with 10% FBS (F101, Vazyme, Nanjing, Jiangsu, China), 1% PS (15140122, Gibco, Grand Island, NY, USA), 10 mM β-glycerol phosphate (G9422, Sigma, St. Louis, MO, USA), and 50 μg/mL L-ascorbic acid (A92902, Sigma, St. Louis, MO, USA). After 14 days, cells were subjected to mineralization staining by 0.1% (w/v) Alizarin Red S (pH = 4.2, A5533, Sigma, St. Louis, MO, USA) for 10 min at room temperature after fixation with 70% ethanol for 5 min. Images were obtained by using a flatbed scanner (V850 pro, EPSON, Suwa-shi, Nagano-ken, Japan). Quantification of mineralized areas was performed using ImageJ software (version 1.52a, NIH, Bethesda, MD, USA) according to well-established protocols [50].

### *Nr1i2* knockdown or overexpression in vitro

To knockdown *Nr1i2* in primary BMSCs, the specific siRNAs containing sequence (5′-CCA CUC AUG CAA GAG UUA UUU TT-3′) targeting mus musculus *Nr1i2* gene (si*Nr1i2*) or sequence (5′-UUC UCC GAA CGU GUC ACG UTT-3′) targeting null as control (siNC) were synthesized by QBiO Technology (Shanghai) Co., Ltd. When cell confluency reached 50–70%, 100 nM siRNA solutions were introduced into the cells by using Lipofectamine™ 3000 Transfection Reagent (L3000075, Invitrogen, Carlsbad, CA, USA) and Opti-MEM (11058021, Gibco, Grand Island, NY, USA) according to the manufacturer’s protocols. To overexpress *Nr1i2* in primary BMSCs, a total amount of 2 μg plasmid containing the full-length coding sequence of mus musculus *Nr1i2* gene (P_*Nr1i2*_) or a vector as the negative control (P_NC_) were transfected into cells by using the above reagents when cells reached 70−90% confluence. Western blot was performed to evaluate the efficiency of *Nr1i2* knockdown or overexpression 72 h after transfection.

### Methylthiazol-yl-diphenyl-tetrazolium bromide (MTT) assay

To explore the viability of primary BMSCs after different treatment, cells were incubated with 0.5 mg/ml 3-(4,5-dimethylthiazol-2-yl)-2,5-diphenyltetrazolium bromide (MTT, M6494, Invitrogen, Carlsbad, CA, USA) in the culture medium at 37 °C for 2 h. Following the removal of the supernatant, formazan crystals were solubilized in 100 μL of dimethyl sulfoxide (DMSO, HY-Y0320C, MedChemExpress, Shanghai, China). Absorbance at 550 nm was measured using a microplate reader (Multiskan FC, Thermo, Rockford, IL, USA), with values to determine relative cell viability.

### Measurement of inflammation factors and intracellular oxidative stress level

Primary BMSCs were transfected with si*Nr1i2* or siNC as control 72 h before the conditional medium and cells was collected. The Il1β, Il6, and Tnfα level in the conditional medium, intracellular level of T-SOD, GSH-PX, MDA, and ROS were determined by using the Mouse IL-1β ELISA Kit (HJ177, Epizyme Biotech, Shanghai, China), Mouse IL-6 ELISA Kit (HJ182, Epizyme Biotech, Shanghai, China), and Mouse TNF-α ELISA Kit (HJ207, Epizyme Biotech, Shanghai, China), Total superoxide dismutase (SOD) assay kit (A001-3-2, Jiancheng, Nanjing, China), Glutathione Peroxidase (GSH-PX) Assay Kit (A005-1-2, Jiancheng, Nanjing, China), Malondialdehyde (MDA) assay kit (A003-1-2, Jiancheng, Nanjing, China), and DCFDA/H2DCFDA-Cellular ROS Assay Kit (ab113851, abcam, Cambridge, UK) according to the manufacturer’s instructions, respectively.

### Molecular docking

The structure of mus musculus Pxr protein and pregnenolone 16α-carbonitrile (PCN) was obtained from the UniProt database (ID: O54915) and PubChem database (ID: 15032), respectively. Molecular docking between the protein Pxr and PCN was performed by using the online Docking tools (https://cadd.labshare.cn/cb-dock2). The visualization of the docking results was done by using PyMOL (version 3.1.5.1, Schrödinger, Inc., New York, NY, USA).

### Reagents

Other reagents used in this study (with vendors not specified above) include: PCN (HY-131723, MedChemExpress, Shanghai, China), administered to animals via intraperitoneal injection at 50 mg/kg/day for 6 weeks; γ-L-glutamyl-L-cysteinyl-glycine (GSH, HY-D0187, MedChemExpress, Shanghai, China), delivered twice weekly for 6 weeks via tail vein injection at a dose of 10 mg/kg; and PI3K/AKT-IN-1 (5 μM, HY-144806, MedChemExpress, Shanghai, China) for in vitro experiment.

### Statistical analysis

The quantitative data are presented as mean ± standard error of the mean (s.e.m.), with n representing the number of animals for in vivo test in each group or the number of independent repeats for in vitro experiments. Sample sizes were determined by preliminary data. Data distribution was tested with the Shapiro-Wilk test. An unpaired two-tailed Student’s t-test was employed for statistical comparisons between two groups, whereas one-way or two-way analysis of variance (ANOVA) with relevant *post hoc* tests was used to assess differences across multiple groups. Scatter plots with bars were used to describe the entire population without assumptions about statistical distribution. Significance levels are denoted as follows throughout the figures: ns *p* > 0.05, *p < 0.05, **p < 0.01, ***p < 0.001. Statistical analyses were performed using GraphPad Prism software (Version 10.2.2, Boston, MA, USA). No experimental subjects (mice), biological samples, or data points were excluded from the final dataset during the study. Analyses were performed by the investigators in an unblinded manner.

## Supplementary information

Supplemental Tables and Figures

Supplemental full and uncropped western blots

## Data Availability

The datasets generated during and/or analysed during the current study are available from the corresponding author on reasonable request.
