# Supplementary material for: Pregnane X receptor protects against age-related bone loss in males via PI3K/Akt-mediated inhibition of apoptosis
Source: Cell Death Discov. 2025 Nov 7;11:511. doi: 10.1038/s41420-025-02797-y (PMC12594874; doi:10.1038/s41420-025-02797-y)
Supplement: Supplementary file 1 — Supplemental Tables and Figures [file 41420_2025_2797_MOESM1_ESM.docx]

**Supplementary materials**

**Pregnane X receptor protects against age-related bone loss in males via PI3K/Akt-mediated inhibition of apoptosis**

Shangzhi Li, Yu Xu, Wenpeng Xu, Dingxin Zhang, Xiangyu Lin, Peijie Hu^**^, Haipeng Si^*^

**Table of Contents**

| Table S1 ················································································ | 2 |
| --- | --- |
| Table S2 ················································································ | 3 |
| Fig. S1 ·················································································· | 4 |
| Fig. S2 ·················································································· | 5 |
| Fig. S3 ·················································································· | 6 |
| Fig. S4 ·················································································· | 7 |

**Supplementary Tables**

**Table S1**. **Primers for qPCR analysis**

| **Genes** | **Forward (5'-3')** | **Reverse (5'-3')** |
| --- | --- | --- |
| Pxr encoding gene *Nr1i2* | | |
| *Nr1i2* | TCTGACTGCAGCTGGTTAGC | TGTTACAGGGTTCGACCTGC |
| Housekeeping gene | | |
| *Gapdh* | AACGACCCCTTCATTGAC | TCCACGACATACTCAGCAC |
| Osteogenic differentiation-related genes | | |
| *Runx2* | GCACAAACATGGCCAGATTCA | AAGCCATGGTGCCCGTTAG |
| *Sp7* | ATGGCGTCCTCTCTGCTTG | TGAAAGGTCAGCGTATGGCTT |
| *Col1a1* | ACCTGTGTGTTCCCTACT | GGTCATGCTCTCTCCAAAC |
| *Alpl* | GTGGAATACGAACTGGATGAG | GTTCCAGACATAGTGGGAATG |
| *Spp1* | AGCAAGAAACTCTTCCAAGCAA | GTGAGATTCGTCAGATTCATCCG |
| *Bglap* | CTGACCTCACAGATCCCAAGC | TGGTCTGATAGCTCGTCACAAG |
| Inflammation-related genes | | |
| *Il1β* | TGCCACCTTTTGACAGTGATG | ATGTGCTGCTGCGAGATTTG |
| *Il6* | GCCTTCTTGGGACTGATGCT | ACAGGTCTGTTGGGAGTGGT |
| *Tnf* | ATGGCCTCCCTCTCATCAGT | TTTGCTACGACGTGGGCTAC |
| Apoptosis-related genes | | |
| *Bax* | CTCAAGGCCCTGTGCACTAA | TTGGATCCAGACAAGCAGCC |
| *Bcl2* | GCGTCAACAGGGAGATGTCA | GCATGCTGGGGCCATATAGT |
| *Casp3* | GAGCTTGGAACGGTACGCTA | CCACTGACTTGCTCCCATGT |

**Table S2. Information of antibodies**

| **Antibodies** | **Cat #** | **Company** | **Applications** |
| --- | --- | --- | --- |
| Primary antibodies | | | |
| Pxr (1:1000) | 67912 | Proteintech (Wuhan, China) | IB |
| Alp (1:1000) | ab229126 | Abcam (Cambridge, UK) | IB |
| Ocn (1:1000) | ab133612 | Abcam (Cambridge, UK) | IB |
| Sp7 (1:1000) | ab209484 | Abcam (Cambridge, UK) | IB |
| Bcl2 (1:1000) | 26593-1-AP | Proteintech (Wuhan, China) | IB |
| Bax (1:1000) | 50599-2-Ig | Proteintech (Wuhan, China) | IB |
| Pro-caspase 3 (1:1000) | R23315 | ZEN-BIOSCIENCE(Chengdu,China) | IB |
| Cleaved-caspase 3 (1:1000) | 25128-1-AP | Proteintech (Wuhan, China) | IB |
| p-PI3K (1:500) | 341468 | ZEN-BIOSCIENCE(Chengdu,China) | IB |
| t-PI3K (1:1000) | 251221 | ZEN-BIOSCIENCE(Chengdu,China) | IB |
| p-Akt (1:500) | R381555 | ZEN-BIOSCIENCE(Chengdu,China) | IB |
| t-Akt (1:1000) | 342529 | ZEN-BIOSCIENCE(Chengdu,China) | IB |
| β-actin (1:5000) | 66009 | Proteintech (Wuhan, China) | IB |
| PE-conjugated anti-CD29  (1μg/10^6^ cells) | 562801 | BD Biosciences (NJ, USA) | FC |
| Alexa Fluor® 647-conjugated anti-CD34 (1μg/10^6^ cells) | 560233 | BD Biosciences (NJ, USA) | FC |
| APC-Cy^TM^7-conjugated anti-CD45 (1μg/10^6^ cells) | 561037 | BD Biosciences (NJ, USA) | FC |
| FITC-conjugated anti-CD90  (1μg/10^6^ cells) | 561973 | BD Biosciences (NJ, USA) | FC |
| Secondary antibodies | | | |
| HRP-conjugated Goat Anti-Mouse IgG(H+L) (1:5000) | SA00001-1 | Proteintech (Wuhan, China) | IB |
| HRP-conjugated Goat Anti-Rabbit IgG(H+L) (1:5000) | SA00001-2 | Proteintech (Wuhan, China) | IB |

**Supplementary Figures**


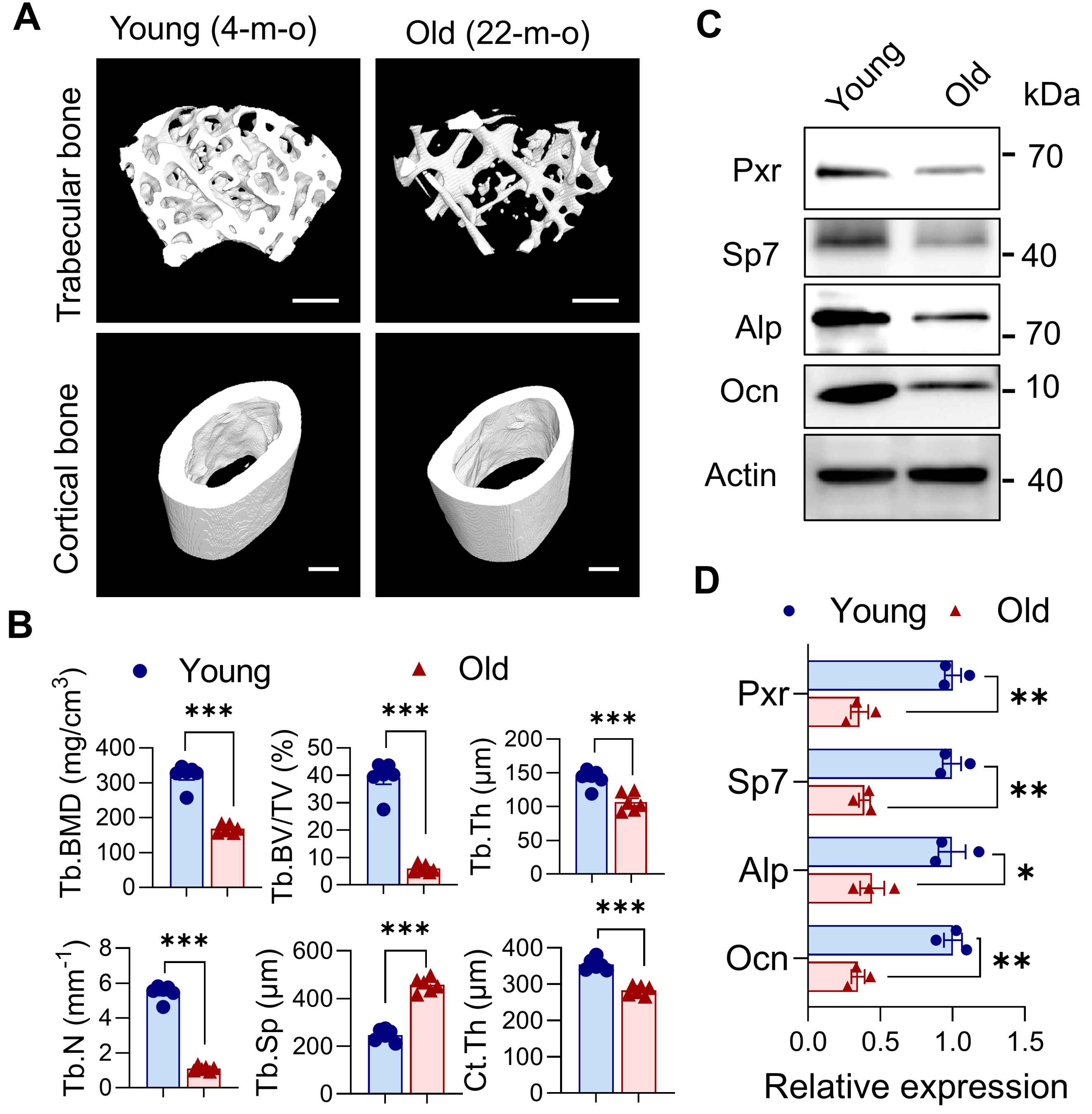


**Fig. S1. The Pxr expression was downregulated with aging. (A-B)** Micro-CT analysis of Tb.BMD, Tb.BV/TV, Tb.N, Tb.Th, Tb.Sp, and Ct.Th in distal end and mid-shaft of femora in young (4-month-old) and old (22-month-old) mice. **(C-D)** Western blot for Pxr and osteogenic-related proteins in young and old mice. Data were means ± s.e.m. n = 6 mice in each group. **p* < 0.05, ****p* < 0.001 by t-test. Scale bars, 500 μm (A).

**
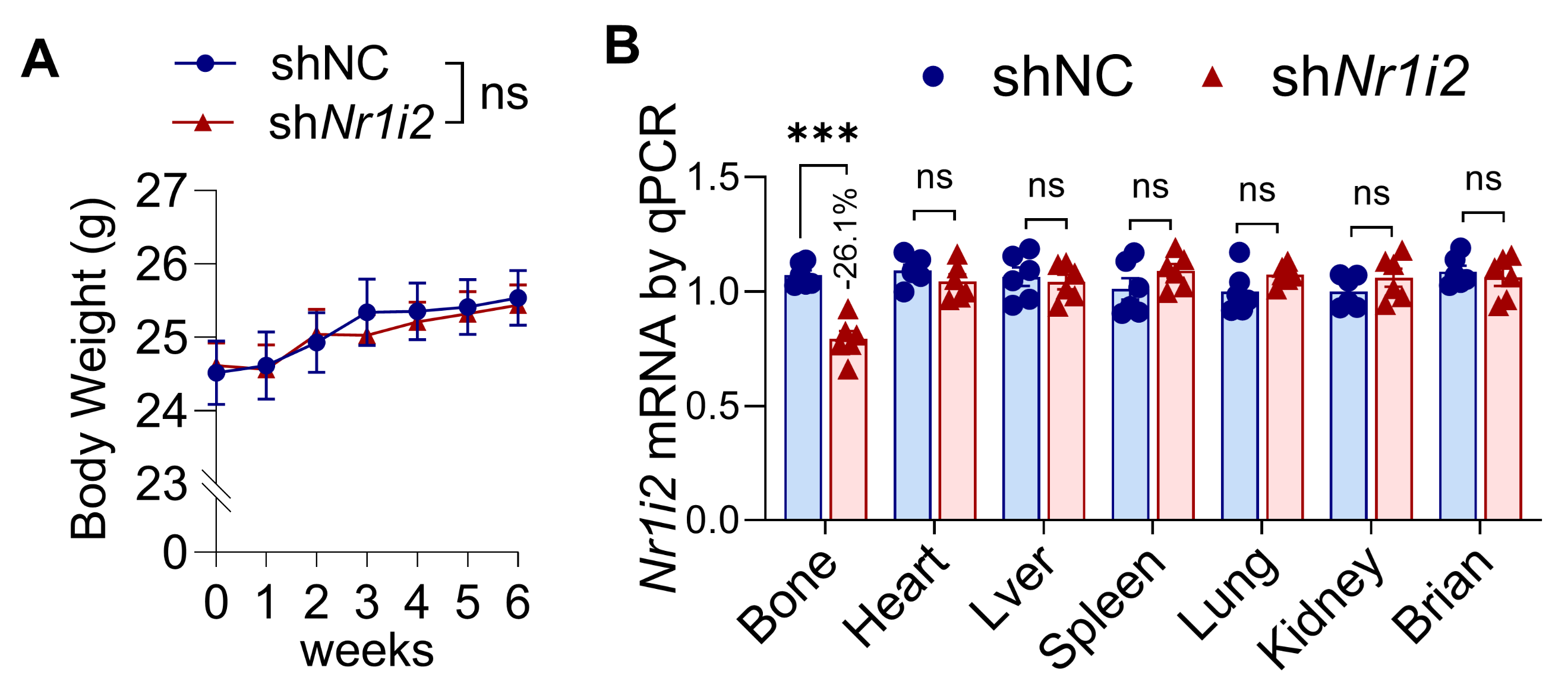
**

**Fig. S2. Knockdown of Pxr in bone *in vivo*. (A)** Record of body weights over the 6 weeks post post AAV shNC and sh*Nr1i2* intramedullary injection. **(B)** qPCR for *Nr1i2* mRNA expression in different tissues at week 3 post AAV intramedullary injection. Data were means ± s.e.m. n = 6 mice in each group. ns *p* > 0.05, ****p* < 0.001 two-way ANOVA (A) or t-test (B).

**
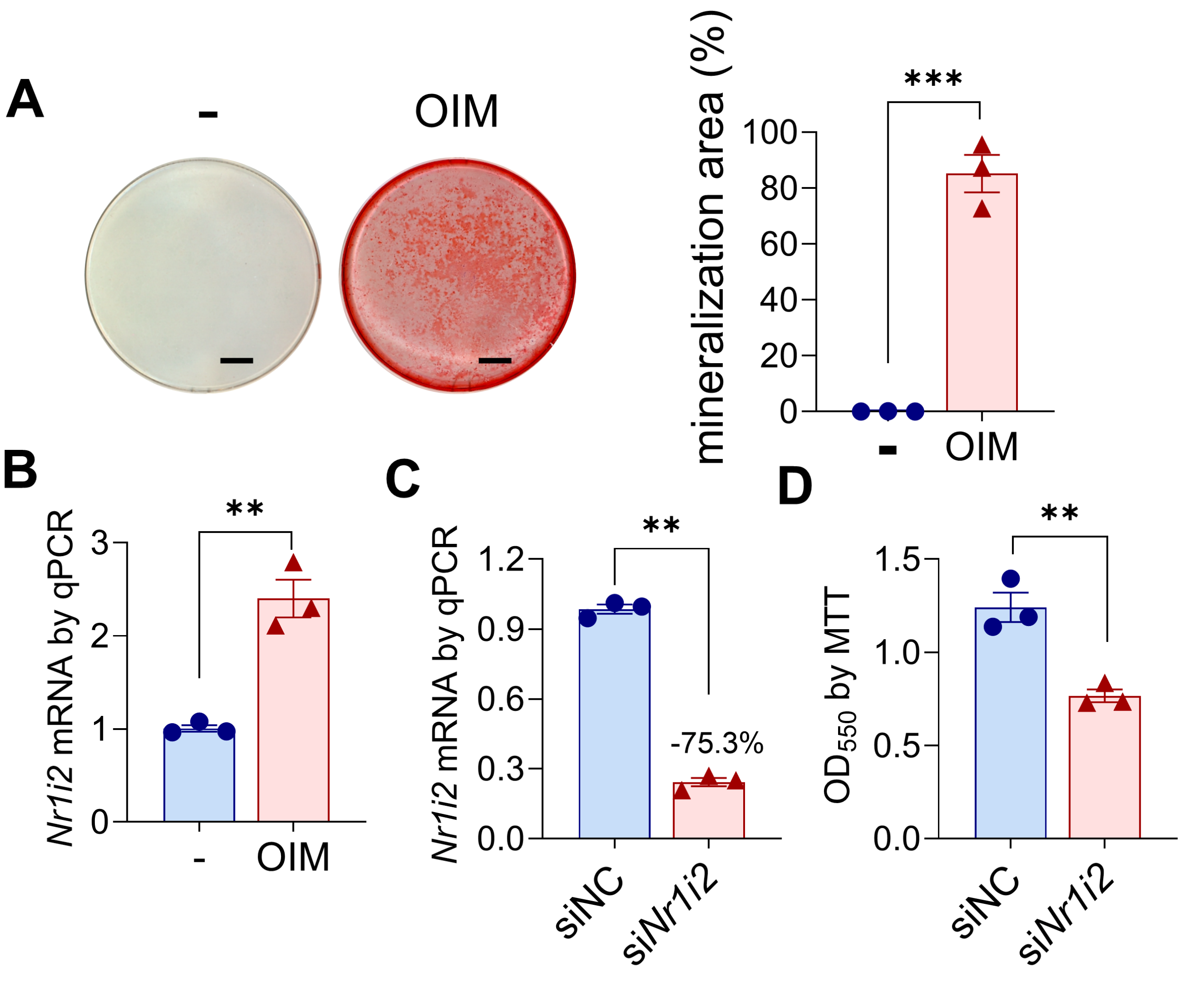
**

**Fig. S3. Knockdown of Pxr in primary BMSCs *in vitro*. (A)** Alizarin Red S staining for mineralization deposits in primary BMSCs with or without osteogenesis induction. **(B)** qPCR for *Nr1i2* mRNA expression in primary BMSCs with or without osteogenesis induction. **(C)** qPCR for *Nr1i2* mRNA in primary BMSCs with or without *Nr1i2* knockdown for 72h. **(D)** MTT assay for primary BMSCs survivability with or without *Nr1i2* knockdown for 72h. Data were means ± s.e.m. n = 3 independent repeats for *in vitro* experiments. ***p* < 0.01, ****p* < 0.001 by t-test. Scale bars, 2.5 mm (E).


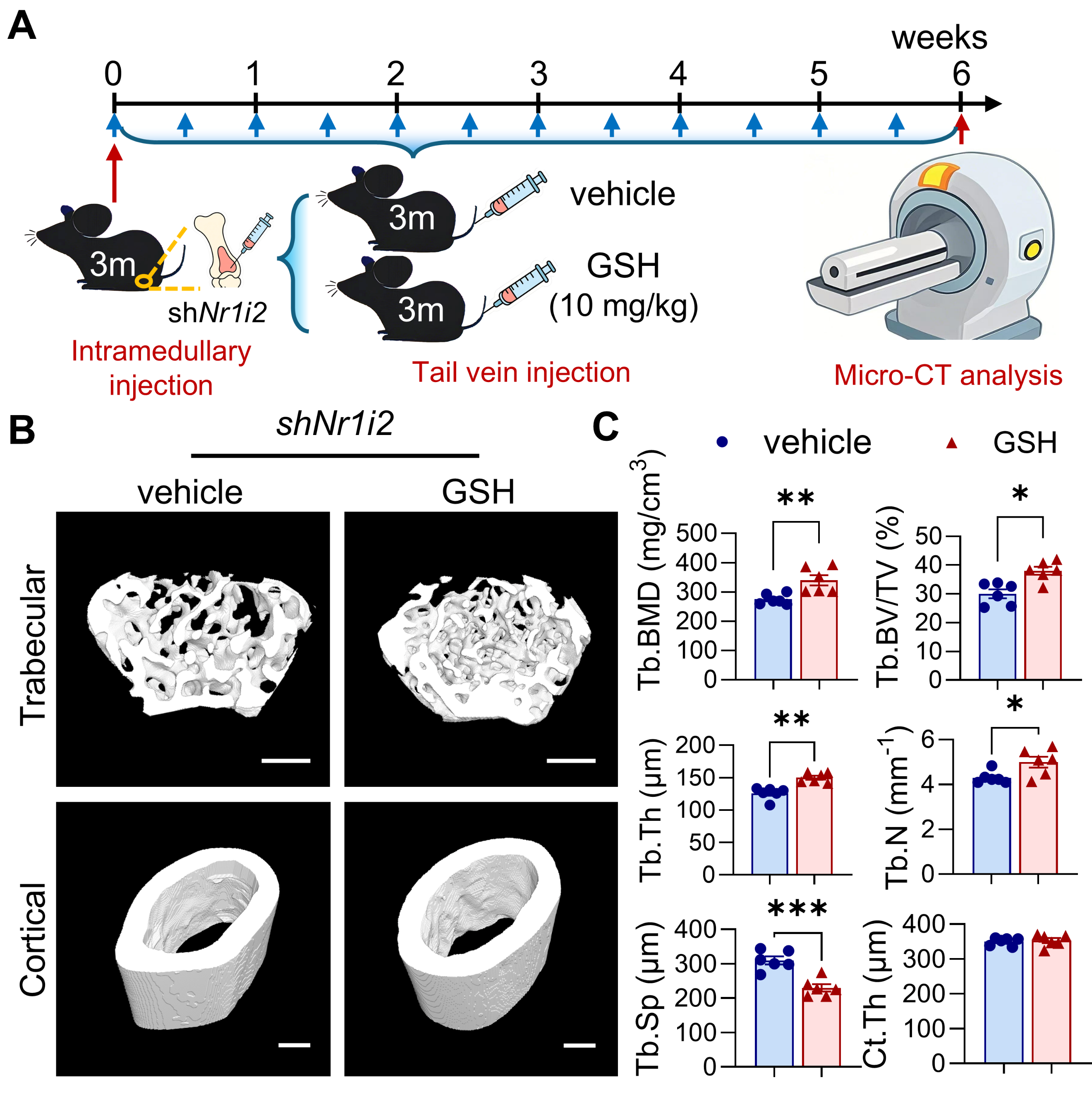


**Fig. S4. The anti-oxidation reagent GSH prevents bone loss in femora with Pxr knockdown. (A)** Schech diagram showing experimental design. AAV medicated Pxr knockdown were performed in week 0 via intramedullary injection. GSH or equivalent amount of normal saline as vehicle control were given to the mice by vein injection twice a week for 6 weeks. Micro-CT was performed in week 6. **(B-C)** Micro-CT analysis of Tb.BMD, Tb.BV/TV, Tb.N, Tb.Th, Tb.Sp, and Ct.Th in distal end and mid-shaft of femora in vehicle and GSH treated mice. Data were means ± s.e.m. n = 6 mice each group. **p* < 0.05, ***p* < 0.01, ****p* < 0.001 by t-test. Scale bars, 500 μm (B).
