# Supplementary figures and images for: Pregnane X receptor protects against age-related bone loss in males via PI3K/Akt-mediated inhibition of apoptosis
Source: Cell Death Discov. 2025 Nov 7;11:511. doi: 10.1038/s41420-025-02797-y (PMC12594874; doi:10.1038/s41420-025-02797-y)

Uncropped images for Blots

Fig. 3D

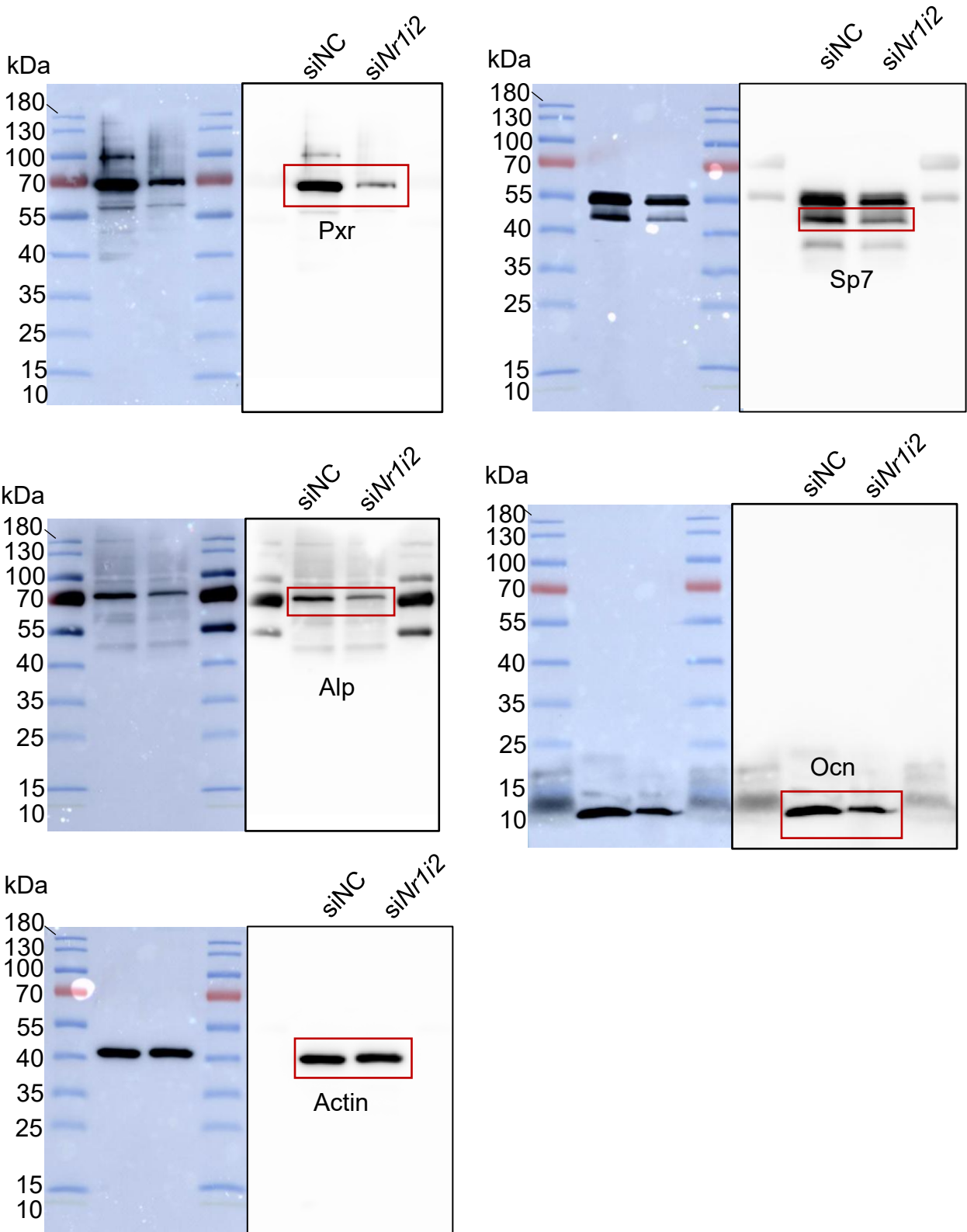

Fig. 4E

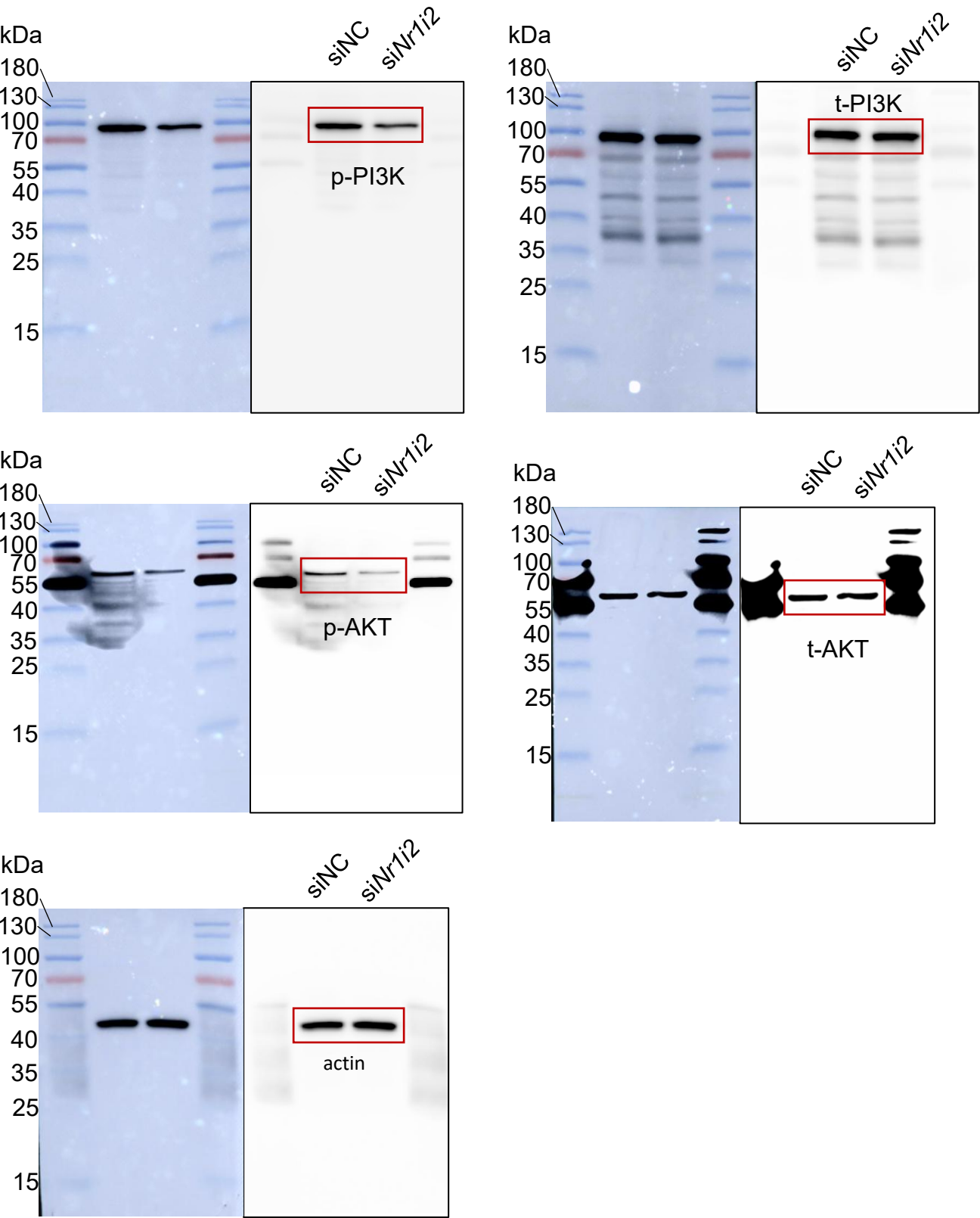

**Fig. 4F**

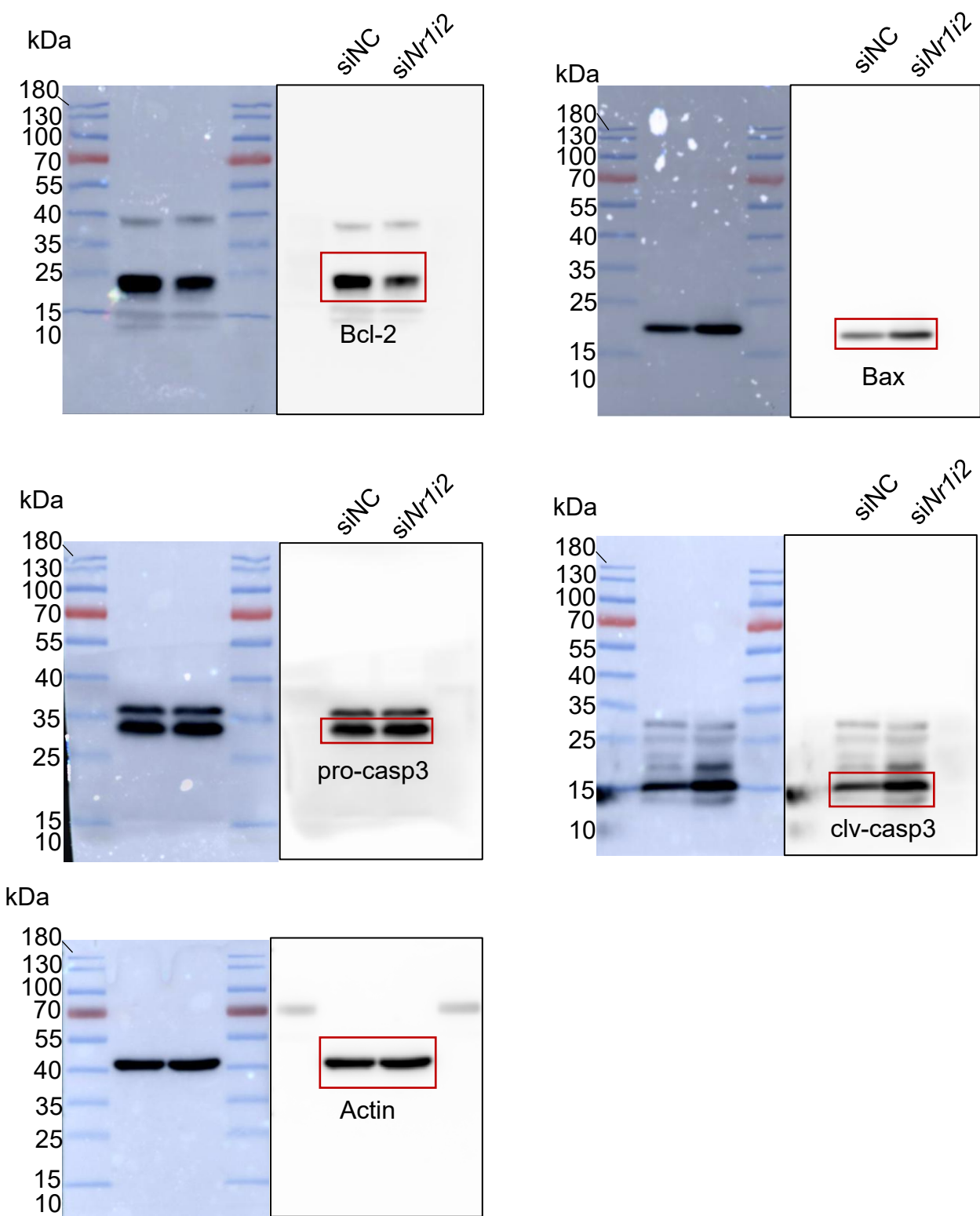

Fig. 5B

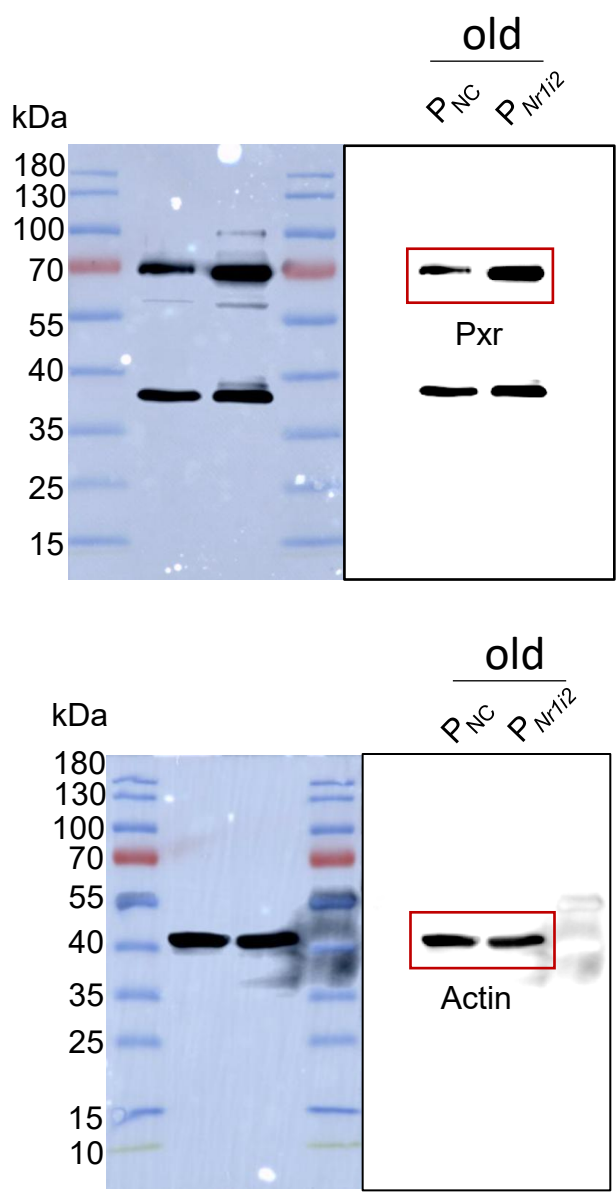

Fig. 5C

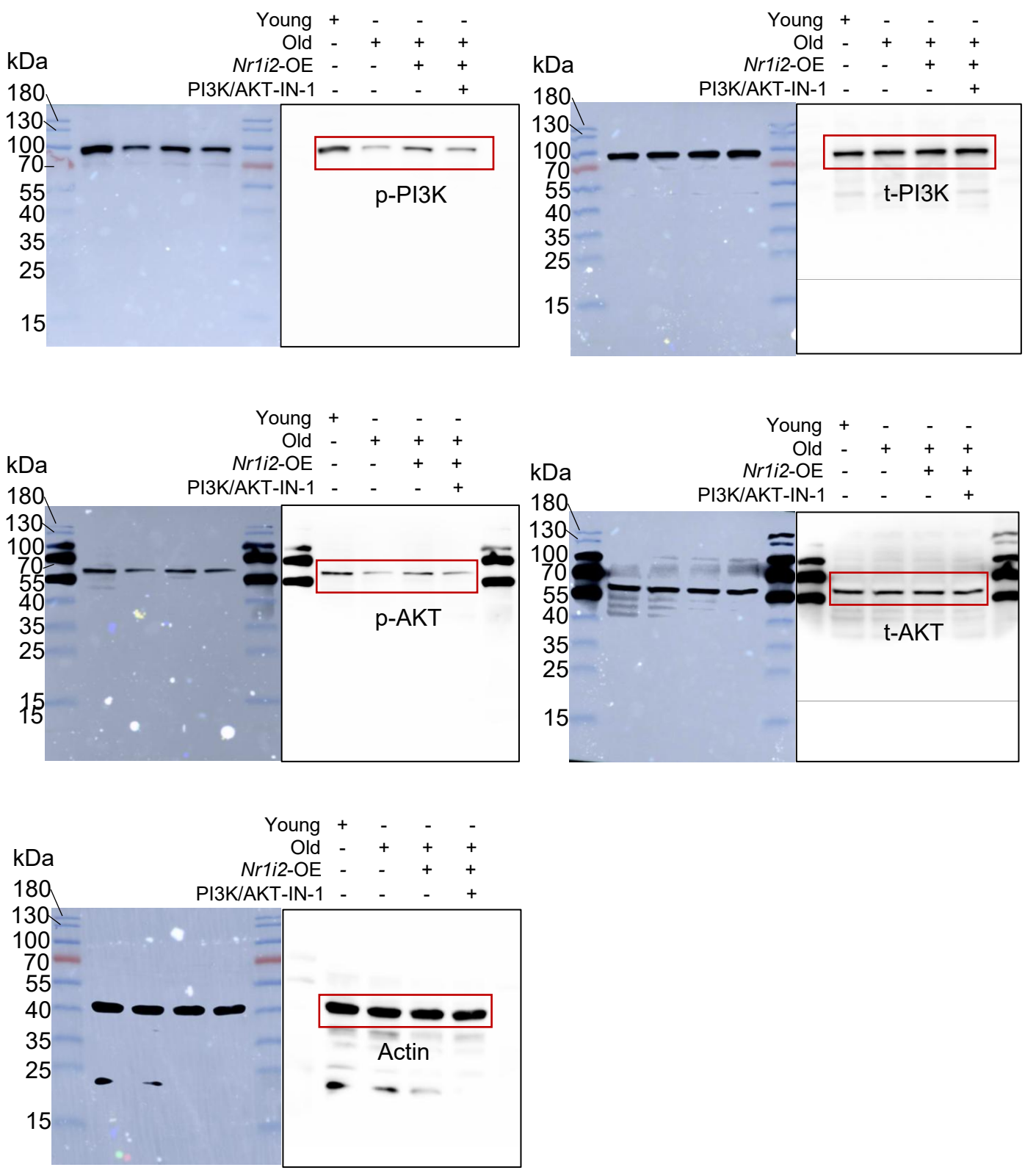

Fig. 5D

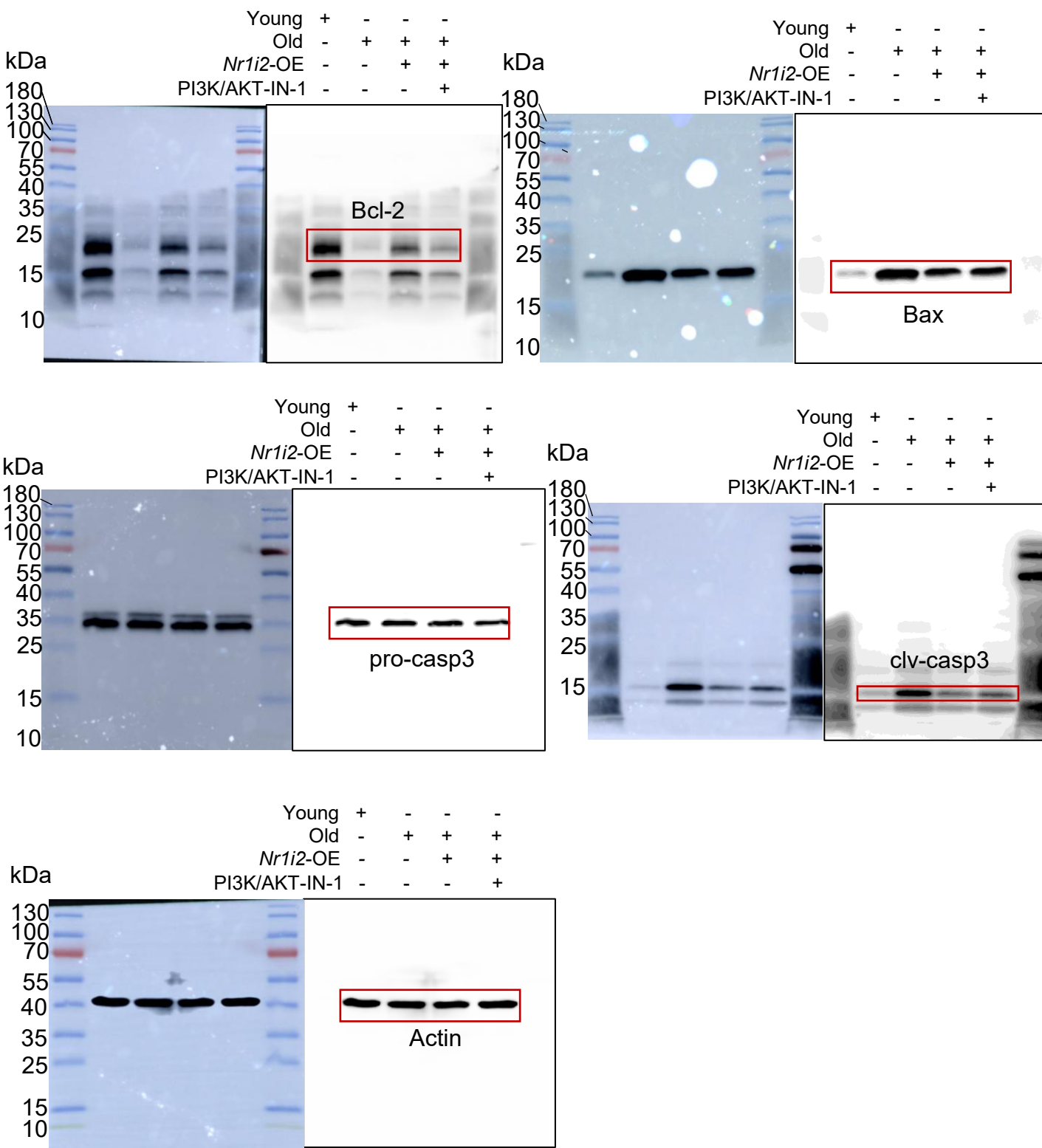

Fig. S1C

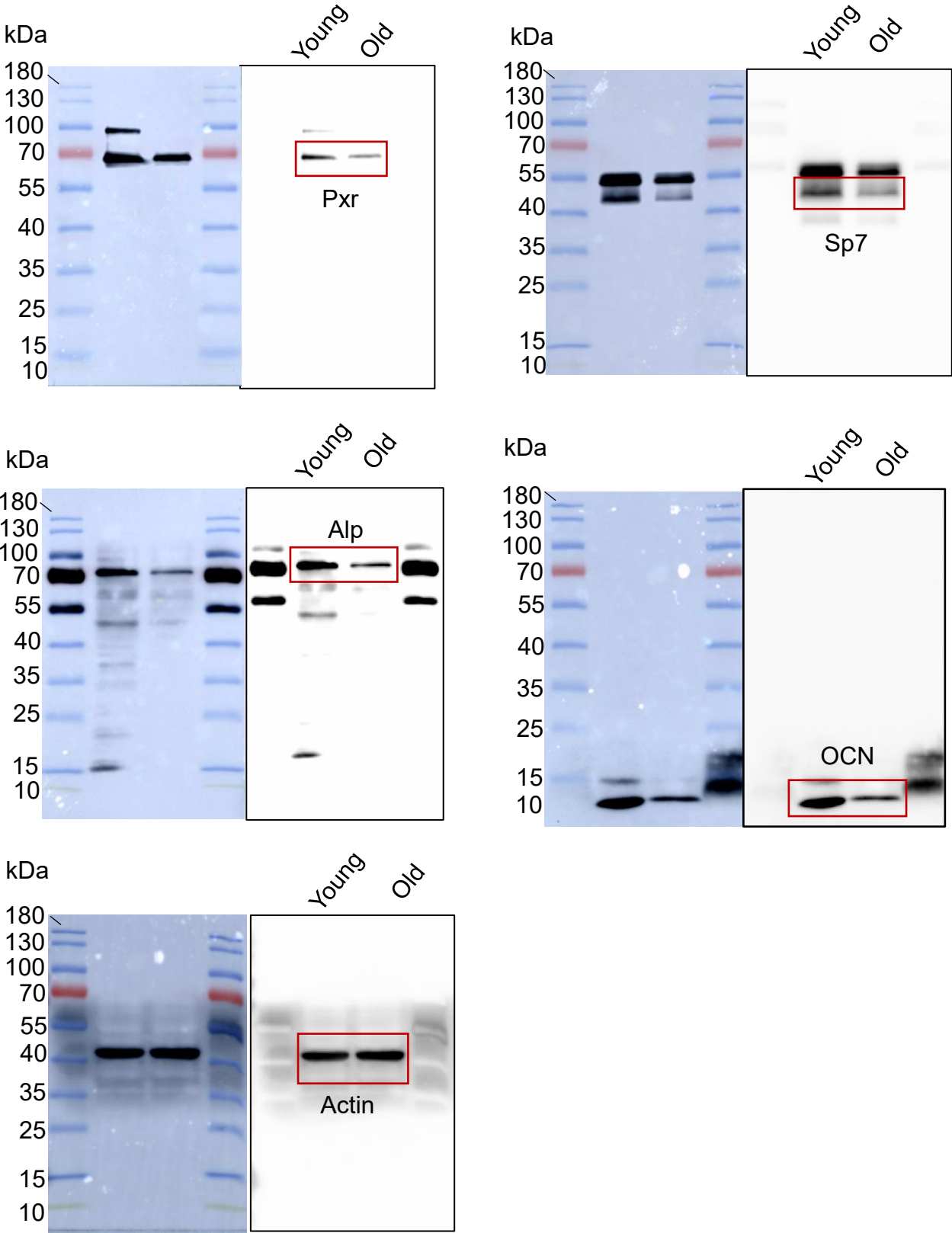

Supplement: Supplementary file 2 — Supplemental full and uncropped western blots [file 41420_2025_2797_MOESM2_ESM.pdf]
